# Phylogenomic inference and demographic model selection suggest peripatric separation of the cryptic steppe ant species *Plagiolepis pyrenaica* stat. rev.

**DOI:** 10.1111/mec.16828

**Published:** 2023-01-20

**Authors:** Philipp Kirschner, Bernhard Seifert, Joelle Kröll, the STEPPE Consortium, Birgit C. Schlick‐Steiner, Florian M. Steiner

**Affiliations:** ^1^ Department of Ecology University of Innsbruck Innsbruck Austria; ^2^ Department of Botany University of Innsbruck Innsbruck Austria; ^3^ Senckenberg Museum of Natural History Görlitz Germany

**Keywords:** demographic inference, Eurasian steppe, integrative species delimitation, morphometrics, pleistocene speciation

## Abstract

The ant *Plagiolepis taurica* Santschi, 1920 (Hymenoptera, Formicidae) is a typical species of the Eurasian steppes, a large grassland dominated biome that stretches continuously from Central Asia to Eastern Europe and is represented by disjunct outposts also in Central and Western Europe. The extent of this biome has been influenced by the Pleistocene climate, and steppes expanded recurrently during cold stages and contracted in warm stages. Consequently, stenotopic steppe species such as *P*. *taurica* repeatedly went through periods of demographic expansion and severe isolation. Here, we explore the impact of these dynamics on the genetic diversification within *P*. *taurica.* Delimitation of *P*. *taurica* from other *Plagiolepis* species has been unclear since its initial description, which raised questions on both its classification and its spatiotemporal diversification early on. We re‐evaluate species limits and explore underlying mechanisms driving speciation by using an integrative approach based on genomic and morphometric data. We found large intraspecific divergence within *P*. *taurica* and resolved geographically coherent western and eastern genetic groups, which likewise differed morphologically. A morphometric survey of type material showed that *Plagiolepis* from the western group were more similar to *P. barbara pyrenaica* Emery, 1921 than to *P. taurica*; we thus lift the former from synonymy and establish it as separate species, *P. pyrenaica* stat. rev. Explicit evolutionary model testing based on genomic data supported a peripatric speciation for the species pair, probably as a consequence of steppe contraction and isolation during the mid‐Pleistocene. We speculate that this scenario could be exemplary for many stenotopic steppe species, given the emphasized dynamics of Eurasian steppes.

## 1 INTRODUCTION

The Eurasian steppe biome covers a vast area from Mongolia in the east to Europe in the west. In its natural extent, this so‐called “Eurasian steppe belt” represents the second largest terrestrial biome in the world (Wesche et al., 2016). As such, the biome also features some of the most species‐rich grassland ecosystems worldwide (Kuzemko et al., 2016) and is of large conservation value (Kirschner et al., 2020). The extent of the Eurasian steppe biome has been very plastic during the Pleistocene climatic oscillations and was much larger than in today's warm stage conditions during Pleistocene cold stages such as the last glacial period (LGP) (Allen et al., 1999; Binney et al., 2017; de Beaulieu & Reille, 1992; Sadori et al., 2016). Climate‐driven and recurring range alterations of entire biomes have been suggested to be important proximate causes of increased speciation by driving isolation and demographic changes of populations (e.g., de Queiroz et al., 2022; Ebdon et al., 2021). This process, often referred to as “Pleistocene species pump” (Haffer, 1969), is particularly evident in biomes that were strongly influenced by the Pleistocene climate oscillations, such as the Eurasian steppes (Kajtoch et al., 2016; Kirschner et al., 2020; Seidl et al., 2021).

The available data on Pleistocene diversification of European ants suggested diversification scenarios that reflected general scenarios postulated for biota of temperate Europe (Hewitt, 1999), that is in short, lineage diversification and formation through allopatric isolation in Southern European refugia during Pleistocene cold stages (e.g., *Leptothorax* Seifert, 1995; *Formica* Goropashnaya et al., 2004; *Tetramorium* Schlick‐Steiner et al., 2007; *Myrmica* Leppänen et al., 2013; *Temnothorax* Csősz et al., 2014). In contrast, recent studies targeting European steppe biota showed significant differences in terms of Pleistocene range dynamics and lineage formation compared with other species from temperate Europe (Frajman et al., 2019; Kajtoch et al., 2016; Kirschner et al., 2020, 2022; Seidl et al., 2021; Záveská et al., 2019). Demographically, steppe biota reacted in an opposite manner to climatic oscillations compared with most temperate species, meaning that they underwent population increases and range expansions in Pleistocene cold stages and contractions in warm stages (Kirschner et al., 2022). Thus, their present‐day distribution reflects their contracted warm‐stage niche, and despite the small size of these refugia and the recurring cold‐stage expansion of Eurasian steppes into Europe, distinct lineages of steppe biota restricted to Europe evolved since the mid‐Pleistocene (Kirschner et al., 2020).

While cross‐taxon studies have enabled a broad and novel understanding of diversification, historical biogeogeography, and demography of steppe biota on a continental scale (Kajtoch et al., 2016; Kirschner et al., 2020, 2022), no study has explored underlying speciation mechanisms in detail or has evaluated taxonomic consequences in this framework. The ant *Plagiolepis taurica* Santschi, 1920 is a suitable arthropod model to do so. *Plagiolepis taurica* is widespread in the Eurasian steppe biome where it occurs exclusively in steppic grassland. Genomic data have suggested that the nominal taxon *P*. *taurica* contains two significantly divergent lineages that split in the mid‐Pleistocene period (Kirschner et al., 2020). This is partly reflected also by morphological incongruences within *P*. *taurica*, which also fuelled a taxonomic dispute that has been going on for several decades.

Central European ants are probably among the best studied in the world (Seifert, 1999, 2018). In the recent past this well studied ant fauna has experienced many taxonomic changes, featuring both the synonymization of taxa (e.g., Csősz, 2012), rank elevations (e.g., Seifert, 2011, 2012a, 2019a) and the description of new species (e.g., Seifert, 2012b, 2019a; Seifert et al., 2017; Steiner et al., 2018; Wagner et al., 2017). As mentioned in the previous paragraph, morphological plasticity has led to a recurring series of taxonomic conflicts such as on the delimitation of *P*. *taurica* from the morphologically very similar *P*. *vindobonensis*. Both taxa were described from steppic habitats in Europe (*P*. *taurica*: southwestern part of the Pontic Steppe, on the Crimean Peninsula [Santschi, 1920], *P*. *vindobonensis*: western edge of the Pannonian basin, close to Vienna [Lomnicki, 1925]). The most important events in their taxonomic history are the synonymization of *P*. *vindobonensis* with *P. taurica* (Radchenko, 1996, 1989), the revival of *P*. *vindobonensis* from synonymy (Seifert, 2007), and ultimately the resynonymization of *P*. *vindobonensis* with *P*. *taurica* (Radchenko, 2016, Figure 1). A recent study even suggested that *P*. *taurica* is a junior synonym of *P*. *pallescens* Forel, 1889 (Salata et al., 2018). While such taxonomic incongruence might only reflect different opinions among describers or methodological flaws, it might also point at an enigmatic evolutionary history. The latter probably applies to the case of *P*. *taurica*.

**FIGURE 1:**
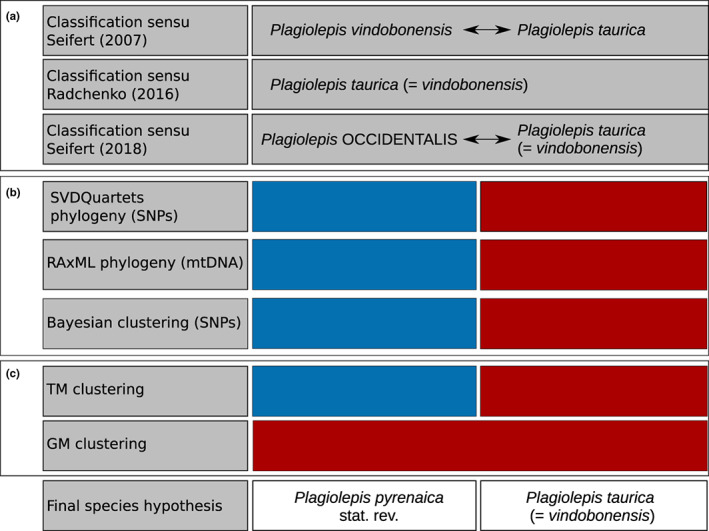
Summary of the taxonomic history of the *Plagiolepis taurica* complex and summary of the results of this study that lead to the final species hypothesis. Following the gene and gene expression (GAGE) species concept (Seifert, 2020a), genomic single nucleotide polymorphisms (SNPs) inferred via restriction‐site associated DNA sequencing and traditional morphometrics (TM) were selected as decisive delimitation criteria. (a) Taxonomic status quo according to Seifert (2007), Radchenko (2016) and Seifert (2018). (b) Results of phylogenetic analyses and Bayesian clustering (Figures 1 and 2a); mtDNA = mitochondrial DNA. (c) Morphometric results; TM, traditional morphometrics; GM, geometric morphometrics (Figure 3)

Combining multiple lines of evidence in elaborating species hypotheses, an approach labelled integrative taxonomy (Schlick‐Steiner et al., 2010), has proven to be powerful in solving taxonomic ambiguities, foremost by taking evolutionary history into account (Dejaco et al., 2016). Integrative taxonomy does not rely on any a priori defined species hypotheses, and therefore data should be analysed in an unsupervised (i.e., hypothesis‐free) way whenever feasible. This approach has been shown to be the best way to address complex taxonomic problems in Palearctic ant species in many instances (Steiner et al., 2018; Wagner et al., 2017). Here, we use such an approach to clarify the relationships within the nominal species *P*. *taurica* applying the gene and gene expression (GAGE) species concept (Seifert, 2020a). For this study, we produced the following types of evidence from ants sampled from large parts of the Eurasian steppe biome: (i) genomic single nucleotide polymorphisms (SNPs) inferred via restriction site associated DNA sequencing (RADseq), (ii) fragments of the mitochondrial cytochrome oxidase subunit 1 gene (COI), and (iii) morphometric data obtained via traditional morphometrics (TM) and (iv) geometric morphometrics (GM).

The evolutionary dynamics between extant lineages of *P*. *taurica* were influenced by the oscillating Pleistocene climate. Specifically, range isolation caused by recurring shifts and size changes of Europe's vegetation zones were important factors propagating lineage formation and speciation, a mechanism that has been commonly termed “the Pleistocene species pump” (Haffer, 1969). While also pre‐Pleistocene diversification of xerophilic insect species has been reported, for example from species in Mediterranean dry habitats (Noguerales et al., 2021), the onset of diversification within *P*. *taurica* has been shown to have occurred significantly later, in the Mid‐Pleistocene (Kirschner et al., 2020). Here, we further explored the mode of lineage formation by supplementing phylogenetic results with demographic modelling and ecological niche models (ENM) for LGP and present climate conditions. We propose a total of three alternative hypotheses, that is, hypotheses of peripatric speciation versus allopatric speciation versus parapatric speciation. Under the peripatric speciation hypothesis, peripheral populations from the Eastern European and Central Asian steppes expanded west during cold‐stage steppe expansion, and a small fraction were isolated in steppe outposts in Central and Western Europe when steppes contracted under warm‐stage conditions. Under this scenario, recurrent gene flow in cold stage conditions must have occurred when speciating entities re‐expanded. Alternatively, under the allopatric speciation hypothesis, speciating lineages in Europe were pushed into warm‐stage refugia isolated by strong geographic barriers (e.g., a mountain chain such as the Alps or large‐scale woodland expansion). Under this scenario, no or very little gene flow would be detectable, as such a barrier would have limited contact between speciating entities when they re‐expanded. Finally, under the parapatric speciation hypothesis, extensive gene flow between speciating entities occurred at all times. This could particularly apply for lineages of Eastern European continuous steppes and lineages from steppe outposts on the Eastern Balkan peninsula that probably existed in spatial vicinity at all times, even in (present‐day) warm‐stage conditions. To distinguish between the outlined hypotheses, we used a predefined set of diversification models (Charles et al., 2018; Portik et al., 2017; Záveská et al., 2021) and tested their likelihood using the diffusion approximation approach implemented in dadi (Gutenkunst et al., 2009). Specifically, we employed models that allowed definition of ancestral and speciating entities, were able to discern between vicariance and young and old founder events, captured the initial size of the speciating entity, and accounted for presence or absence of gene flow. Lineage‐specific ENM for warm‐ and cold‐stage conditions enabled us to investigate if niche dynamics are in line with the expansion and contraction scenario postulated for Eurasian steppes in general, and if niches overlapped in different climatic conditions. A statistical comparison of these ENM further allowed us to test if the speciating entities occupy different ecological niches.

## 2 MATERIALS AND METHODS

### 2.1 Material analysed

Ant nests from 61 localities were sampled between 2014 and 2016 from steppe habitats located all over the Eurasian steppe belt (for toponyms used in this manuscript, see Figure S1; localities in Supporting Information Data S1). When a nest was considered to be sufficiently individual‐rich to survive the removal of worker ants, between 20 and 30 workers were removed and conserved in 99% ethanol for later analyses. The distance between nests within one locality never exceeded 2 km. For TM analyses, additional material from the collection of the Senckenberg Museum of Natural History Görlitz (Görlitz, Germany) and type material from various other collections were examined (Appendix A).

A detailed description of the taxonomic position of *P*. *taurica* within west‐palearctic *Plagiolepis* taxonomy, detailed morphometric information on the examined material, and an exhaustive list of potential synonyms are provided in Appendix A.

### 2.2 Restriction site associated DNA data

The restriction site associated DNA sequencing (RADseq) data for *P*. *taurica* have been generated by Kirschner et al. (2020) and are available from the NCBI short read archive (SRA numbers are given in Supporting Information Data S1). Single nucleotide polymorphisms (SNPs) from these raw data were called de novo, using the denovo_map.pl pipeline implemented in Stacks version 2.3 (Catchen et al., 2013). This software was run using the parameters ‐m 5 (minimum number of raw reads to create a stack), ‐n 3 (maximum number of nucleotide mismatches among loci to be considered as orthologous across multiple samples) and ‐M 3 (maximum number of nucleotide mismatches between two stacks in a locus in each sample, Catchen et al., 2013). Parameter optimization was done using a subset of the data and by following the routine proposed by Paris et al. (2017). The SNP data sets were exported from the Stacks catalogue via populations.pl, whereas the write_random_snp flag was implemented in all instances to avoid violating the assumption of site independence (Catchen et al., 2013). For Bayesian clustering analyses in STRUCTURE (Pritchard et al., 2000), only loci that were present in at least 60% of all individuals were exported using the ‐R 0.6 flag in populations.pl (Catchen et al., 2013). For phylogenetic tree inference in SVDQuartets (Chifman & Kubatko, 2014), SNPs were exported in vcf format, and subsequently loci that were missing in more than 50% of all individuals (‐‐max‐missing 0.5) or had a mean sequencing depth below 5 (‐‐min‐meanDP 5) were excluded using vcftools (Danecek et al., 2011). These filtered vcf files were then converted to nexus format using vcf2phylip.py (available at github.com/edgardomortiz/vcf2phylip). The same vcf file was also used as basis for demographic model testing.

### 2.3 Mitochondrial DNA data

The partial fragments of the mitochondrial cytochrome oxidase 1 (COI) gene analysed in this study were generated by Kirschner et al. (2020). All sequence data are available from the NCBI GenBank (accession numbers in Supporting Information Data S1). In addition, available COI sequences of the following European *Plagiolepis* species were downloaded from NCBI GenBank (accession codes in parentheses): *P*. *schmitzi* (MW243655, MW243656), *P*. *pallescens* (MW246082, MW246084‐MW246087), *P*. *xene* (MW243625‐MW243637), and *P*. *pygmaea* (MT606368‐MT606371, MW240958, MW243613‐MW243624); the paleotropical species *P*. *alluaudi* (accession DQ176134) was used as an outgroup.

### 2.4 Phylogenetic analyses and Bayesian clustering

The SVDQuartets method for phylogenetic inference was used to compute phylogenetic relationships between populations based on genome wide SNPs under a multispecies coalescent model (Chifman & Kubatko, 2014). All samples from a given sampling site were defined as populations via a taxset block in the nexus file and individual sequences were correspondingly assigned. SVDquartets was run evaluating all quartets, and 100 nonparametric bootstrap replicates were done to assess robustness of the tree topology.

ML trees based on the mitochondrial COI gene were calculated in RAxML (Stamatakis, 2014) under a GTR model using substitution rates optimized under a gamma rate heterogeneity model (GTRGAMMA); 1000 bootstrap replicates were performed for the best‐scoring tree. Nodes that were supported by more than 75% of the bootstrap replicates were considered to be robust (Hillis & Bull, 1993). The same settings were used to infer the phylogeny based on the available COI gene from other European *Plagiolepis* species. The assignment of populations into *K* groups was inferred via Bayesian clustering as implemented in STRUCTURE version 2.3.4 (Pritchard et al., 2000) using the admixture model and default settings. Ten replicate runs each were done for *K* = 1 to *K* = 5 using a burnin of 100,000 iterations followed by 1000,000 additional MCMC iterations. A hierarchical clustering approach was employed for *K* = 2 to explore substructuring of each cluster at the highest hierarchical level (Janes et al., 2017).

### 2.5 Demographic modelling

To test which demographic model describes the revealed patterns best, the diffusion approximation method implemented in dadi was used (Gutenkunst et al., 2009). Dadi relies on information from the joint site frequency spectrum (jSFS) that summarizes the joint distribution of site frequencies between populations (here always two populations). The folded jSFS for the respective population pairs were inferred from vcf files using the software easySFS (available at github.com/isaacovercast/easySFS). This software was used to downproject the jSFS to account for missingness in the SNP table while at the same time retaining a large number of SNPs. The optimization routine implemented in the two populations' dadi pipeline (Portik et al., 2017) was finally used to test which model from a set of predefined diversification models had the best fit (Figure S2 Charles et al., 2018; Záveská et al., 2021). To evaluate the hypotheses formulated in the introduction, the models included represent three categories of diversification scenarios, and either captured vicariance scenarios, founder events or old founder events. For each of the three categories, additional models capturing gene flow, isolation and changes of effective population size (*N*_e_) were included (Figure S2). All models account for directionality in the initial divergence process and assume the existence of an ancestral (“mainland”) and derived (“island”) population, whereas the ancestral population's fraction present in the derived never exceeds 50% (Charles et al., 2018). Four rounds of optimizations were employed for each model using 60, 70, 70, and 80 replicates, and the model with the highest log‐likelihood was taken for comparison among models. The best performing model was finally selected based on the Akaike information criterion (AIC), and Akaike weights (ω_i)_ were calculated to evaluate the relative likelihood of models in a multimodel regime.

The entities (=populations) for these 2D models were defined based on SVDQuartets phylogenies and Bayesian clustering, and a hierarchical approach was employed that included three 2D model optimizations. At first, the peripatric‐speciation hypothesis was tested assuming *P*. *taurica* to be the ancestral (“mainland”), and *P*. *pyrenaica* stat. rev. the speciating (“island”) entity. In a second step, two more optimization runs were done for *P*. *pyrenaica* stat. rev. and the *P*. *taurica* Balkanic‐Pannonian subgroup, and *P*. *pyrenaica* stat. rev. and the *P*. *taurica* Central Asian‐Pontic subgroup. This was done to test if gene flow between populations occurred only periodically after an initial period of isolation. In both cases, the diversification models published in Portik et al. (2017) were used.

### 2.6 Traditional morphometrics

Traditional morphometric (TM) measurements were done on mounted and dried specimens. Details on the measurement setup used were provided by (Seifert, 2019b). In total, 21 morphological features were measured from at least two specimens per nest of a total of 70 nests from 44 localities (details in Supporting Information Data S1). In addition, specimens from another 30 nests comprising type specimens, and specimens from the collection of the Senckenberg Museum of Natural History Görlitz (Görlitz, Germany) were included (Supporting Information Data S1). The morphological features measured have been successfully used in ant morphometrics (Seifert, 2018; definitions in Table S1).

A morphological species hypothesis was generated via two independent approaches, nest centroid (NC) clustering and partitioning algorithm based on recursive thresholding (PART), similar to the approach described in Csősz and Fisher (2016a, 2016b) and (Seifert, 2019b). NC clustering uses discontinuities in continuous morphometric measurements to group similar cases into clusters. Specifically, the dimensionality in the data was reduced via cumulative linear discriminant analysis (LDA) while using specimens from the same nest as a priori defined entities (Seifert et al., 2013). After this step, scores from the LDA were summarized into a distance matrix and were used to draw a dendrogram using the ward.D2 agglomeration criterion (squared dissimilarities). This procedure was done in R using the packages cluster (Maechler et al., 2021) and MASS (Venables & Ripley, 2002).

In the PART approach, the number of clusters contained in a data set was estimated based on recursive application of the Gap statistic (Tibshirani et al., 2001). Based on the defined stopping thresholds for group size or number of clusters K, the method identifies clusters on multiple hierarchical levels. PART was applied via the part function in the R package clusterGenomics (Nilsen et al., 2013); the minimum cluster size was set to minSize 5, and the maximum number of *K* was set to Kmax 8. Part was done utilizing the hclust and kmeans clustering methods with 1000 bootstrap replicates each. The PART clusters were mapped on the NC dendrogram as coloured bars via the function mark.dendrogram implemented in the R package hyperSpec (Beleites & Sergo, 2020).

### 2.7 Geometric morphometrics

At least two workers per nest from a total of 92 nests sampled from 61 localities were selected for geometric morphometric analyses (Supporting Information Data S1). All specimens were mounted on cardboard, and antennae were removed and preserved before photography. A Leica Z6 APO macroscope using a Leica MC190 HD camera (5.0 x lens, 16.0 x ocular, 2.5 x zoom) was used for photography. From each ant head, between 45 and 65 images were taken from multiple focal planes that were previously defined using Leica Application Suite version 3.7 (Leica Microsystems CMS GmbH, www.leica‐microsystems.com) and automatically focussed via a motorized focus wheel. To avoid blurring, exposure times were adjusted to be below 1 s. The resulting images were merged into a single image using Helicon Focus version 5.1.8 (Helicon Soft Ltd, www.heliconsoft.com).

Each head was photographed in a frontal view in a standardized way. This standardized position was achieved via adjusting focal planes according to characteristic features. Such features were a triangle of reference points on the ants' head, the structures lateral of the compound eyes, reference points on the medial, innermost point of the eyes, and, finally, reference points at the lateral, outermost point of the clypeus. Furthermore, the posterior end of the head was set to appear as straight as possible. A subset of 46 specimens were independently positioned and photographed twice (Supporting Information Data S1). Based on these observations, a Procrustes ANOVA was done to assess the imaging error that could be caused by poor positioning.

All photographs were summarized in a tps file in random order using tpsUtil32 version 1.70 software (Rohlf, 2015). A total of 33 landmarks were placed on the ant heads using tpsDig232 version 2.26 (Rohlf, 2015; Figure S3).

Data analysis was done in MorphoJ version 1.06d (Klingenberg, 2011). Landmark data were aligned via Procrustes transformation along principal axes. To assess the imaging error, a Procrustes analysis of variance (ANOVA) was done for the subset of specimens that were positioned and photographed twice. Based on the Procrustes distance data of all specimens, a covariance matrix was generated to inform a principal component analysis (PCA) and a canonical variate analysis (CVA). For the latter, a‐priori grouping was defined according to the species hypothesis obtained from phylogenetic analyses.

### 2.8 Species concept and delimitation criteria

We used the gene and gene expression (GAGE) species concept (Seifert, 2020a) that stresses nuclear genes and morphological characters to be the most informative character systems to delimit taxonomic entities. In case of this study, genomic RADseq SNPs and morphology (TM & GM) were accordingly chosen as decisive character systems for species delimitation.

Additional evidence, that is, ecological niche models based on the species hypotheses supported by RADseq and mitochondrial DNA, was taken into account to obtain additional information on speciation mode and evolutionary history but was not used as decisive delimitation criterion.

Specifically, RADseq SNPs were used to infer phylogenetic trees and to analyse admixture between the sampled populations. Reciprocal monophyly of phylogenetic groups, the lack of significant admixture between the clusters, and their geographic distribution were used as criteria in defining entities in the final genetic species hypotheses. The final morphological species hypotheses were formed according to the methodology described by Seifert et al. (2013), Csősz and Fisher (2016a), and Seifert (2019b). Three variants of the exploratory data analysis NC‐clustering were applied, and hypotheses were formed by majority decision of these three exploratory data analyses.

The final genetic and final morphological species hypotheses were compared following the recommendations of the GAGE species concept. Priority was given to indication by genomic RADseq data in samples with differing genetic and morphological classifications.

### 2.9 Ecological niche modelling

**FIGURE 2:**
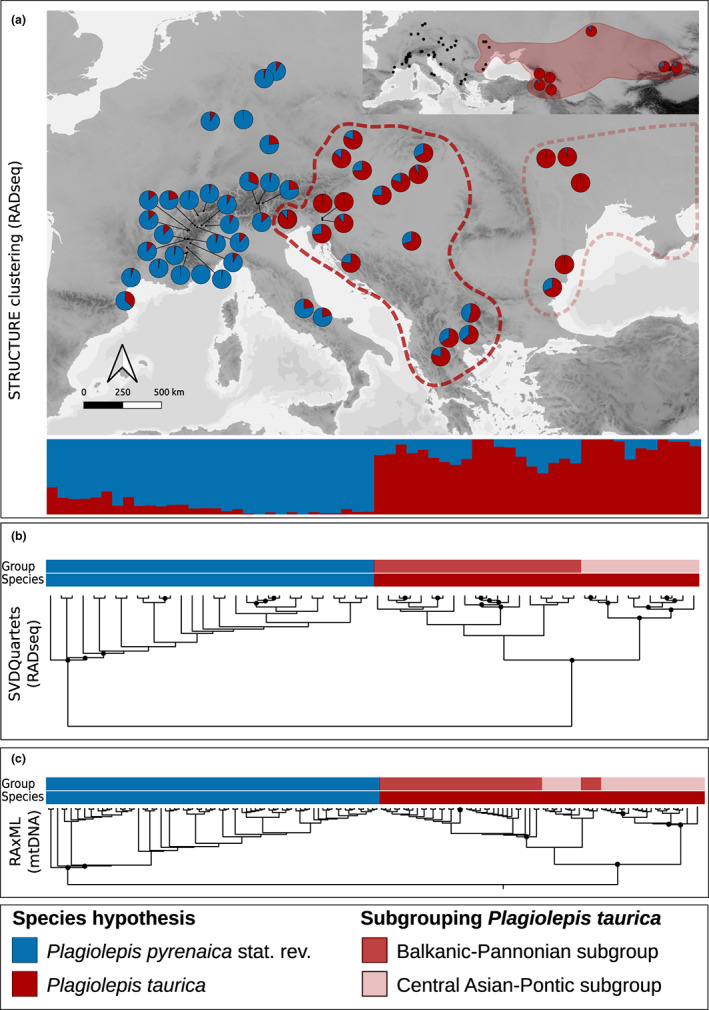
Summary of phylogenetic analyses and Bayesian cluster analyses. Colours in figure legend indicate the final species hypotheses and subgroupings. (a) Geographic distribution of gene pools inferred via Bayesian clustering; cluster assignment and proportion of admixture are shown as coloured pie charts and barplots (based on *K* = 2). Dashed polygons in the map indicate subgroupings and the inset at the upper right corner shows populations in the Caucasus and Central Asia. Each pie and each bar represent one population. Bars are ordered according to the branches in Figure 2b. (b) Phylogenetic tree based on genomic SNPs. Nodes that received over 75% bootstrap support are marked with black circles. Branches correspond to the barplots in Figure 2a. Bars above the tree indicate subgrouping according to the figure legend. (c) Maximum likelihood tree based on mitochondrial DNA. Nodes that received over 75% bootstrap support are marked with black circles. Bars above the tree indicate species hypotheses

Ecological niche modelling was done based on a priori obtained information from genetic data and was thus not performed in a hypothesis‐free manner. Specifically, separate niche modelling runs were done using occurrences of *P*. *taurica* and occurrences of *P*. *pyrenaica* stat. rev. as suggested by phylogenetic analyses (SVDQuartets and COI based ML trees; Figure 2b,c; occurrence data in Supporting Information Data S1).

Before modelling, spatial records of each species were thinned by removing occurrences within a spherical distance of 25 km of an occurrence record to avoid spatial autocorrelation, using spThin in R (Aiello‐Lammens et al., 2015). Also, the distant and sparse occurrences east of the Pontic steppe were excluded to avoid model overfitting. Finally, 22 occurrence records per species were available for niche modelling. Bioclim layers at a resolution of 30 arc‐seconds were obtained from Worldclim version 1.4. (http://www.worldclim.org/), and the extent of these layers was cropped to fit the study region. Due to poor precision of Worldclim precipitation variables in mountainous areas, interpolated precipitation layers of the European Alps, similar to those used in Kirschner et al. (2020), were used instead of the standard precipitation layers. This interpolation was not available for the bio18 and bio19 layers which were consequently excluded. Correlation between layers was assessed using the raster.cor.matrix and raster.cor.plot functions in the R package ENMTools (Warren et al., 2019), and layers with a Pearson correlation coefficient above 0.9 were removed; when layers were to be removed, the respective layer to be retained was selected based on expert knowledge of the biology of Eurasian steppe biota. All models were generated in MAXENT version 3.4.1 (Phillips et al., 2006) using a final set of 11 Bioclim layers (bio2‐4, bio8‐12, and bio15‐17). Regularization parameters and feature classes for these models were evaluated via the R package ENMeval (Muscarella et al., 2014) using jackknife cross validation (*k* = *n*). Model parameters resulting in the largest predictive performance indicated via the area‐under‐the‐curve (AUC) value, and lowest omission rates were selected for the final models. Low omission rates are an indicator that the model is not strongly overfit. When these parameters were equal, model selection was done in favour of the model with fewer parameters.

Niche models were also projected to last glacial maximum (LGM) climate conditions represented by the CCSM4 layers at a 30 arc‐seconds resolution (Schmatz et al., 2015). These projections were done in MAXENT version 3.4.1 (Phillips et al., 2006) using clamping to restrict the range of past variables to the variable range encountered in present‐day model space. Suitable habitats under LGM conditions were defined as those grids that exceeded the 10th percentile training present (P10) threshold of the model and were not covered by glaciers.

A background test between the present‐day niches of each modelled species was done to test for niche divergence (Warren et al., 2008). The area from which background points were sampled was defined as a circular buffer of 50 km around each occurrence point. This area should be selected reflecting the species' dispersal ability (Soberón & Peterson, 2005). Active dispersal over large distances is rare in smaller insects such as *Plagiolepis* ants (Stevens et al., 2014). Further, intranest sibling mating, a reproductive mode in which alates do not even leave their nests, is frequent in *Plagiolepis* ants (Thurin et al., 2011). Considering these life history traits, the dispersal distance will probably not exceed the chosen background. Background tests were done in R using the package ENMTools (Warren et al., 2019) using 100 replicate models and 10,000 randomly chosen background points per run. The null hypothesis of this test was that niche models based on two sets of occurrences are as similar as models based on their environmental background. If the observed niche overlap of the replicated models was significantly higher (=niche conservatism) or lower (=niche divergence) than the empirically inferred niche similarity, this null hypothesis was rejected (alpha = 0.05; Warren et al., 2008).

## 3 RESULTS

### 3.1 Sequencing results

After SNP calling, quality filtering, and the preparation steps for the corresponding analyses, 40,991 SNPs were available for phylogenetic inference in SVDQuartets, 8534 SNPs for Bayesian clustering. The length of the mitochondrial COI gene fragment was 614 bp after trimming (Supporting Information Data S1).

### 3.2 Phylogenetic analyses and genetic clustering

A total of 142 individuals was included for SNP‐based phylogenetic analyses in SVDQuartets representing 60 localities. For analyses in RAxML based on mtDNA, 144 individuals representing 56 localities were analysed. SVDQuartets resolved a split into two main groups that received large bootstrap support (bs; SNP informed SVDQuartets tree: 100%; COI‐informed ML tree: 100%; Figure 2b,c, Figure S4). Our data also showed that both species are geographically consistent in their distribution range and corresponded to the morpho‐species *P*. *taurica* and *P*. *pyrenaica* stat. rev. (Figure 2a). Specifically, we found that *P*. *pyrenaica* stat. rev. is distributed in the Alps, Southern France, the Apennine Peninsula, and Central Germany. All populations from the South‐eastern Alps, the Balkan peninsula, and the western margin of the Pannonian Basin eastward belonged to *P*. *taurica*. Tree topology within *P*. *pyrenaica* stat. rev. showed no distinct structuring with the exception of the most basal nodes that marked the split of populations from Southern France (only SNP phylogeny) and the Apennine Peninsula (SNP & COI phylogenies). The SNP‐informed SVDQuartets tree further supported geographically consistent and distinct subgroups within *P. taurica* (Figure 2b). The most prevalent and well supported subgrouping (bs 100%) separated a group consisting of all Pannonian and Western Balkanic Populations (from here on “Balkanic‐Pannonian subgroup”) and a group containing populations from Central Asia, the Caucasus, and the Pontic steppes of Eastern Europe (from here on “Central Asian‐Pontic subgroup”) (Figure 2b). This subgrouping was not fully resolved in the COI ML tree (Figure 2c), as populations from the Pontic steppes were nested within the Balkanic‐Pannonian subgroup. The COI‐based ML phylogeny including related European *Plagiolepis* species showed that *P*. *taurica* and *P*. *pyrenaica* stat. rev. are a monophylum (bs 96.9%), and *P*. *pallescens* and *P*. *schmitzii* were sister lineages (bs 91%; Figure S5). Two COI sequences from *Plagiolepis* ants from Corsica and Southern France (Degueldre et al., 2021; accessions MW246086, MW246085) were listed as *P*. *pallescens* in NCBI genbank but clustered with *P*. *pyrenaica* stat. rev. in our phylogeny. These individuals clearly belong to *P*. *pyrenaica* and their classification reflects the nomenclatural incongruence owed to the obsolete synonymizations within the complex presented by Salata et al. (2018).

The genetic clusters inferred via Bayesian clustering further reflected the phylogenetic results (*K* = 2 with similar grouping as in the SNP‐ and mtDNA‐informed phylogenetic analyses, Figure 2a). Populations with significant admixture were frequent in the Pannonian basin, on the Balkan peninsula (especially in the South), on the Apennine Peninsula, and in Southern France but less so in the Alps and in Central Germany (in the case of *P*. *pyrenaica* stat. rev.) or from the Carpathians eastward (*P*. *taurica*). The hierarchical subclustering of *K* = 2 clusters showed no distinct substructure within *P*. *pyrenaica* stat. rev., but resolved subgroups within *P*. *taurica*. Specifically, the Balkanic‐Pannonian subgroup and the Central Asian‐Pontic subgroup was resolved similar to the SVDQuartets phylogeny (Figure 2a, Figure S6).

### 3.3 Morphometrics

**FIGURE 3:**
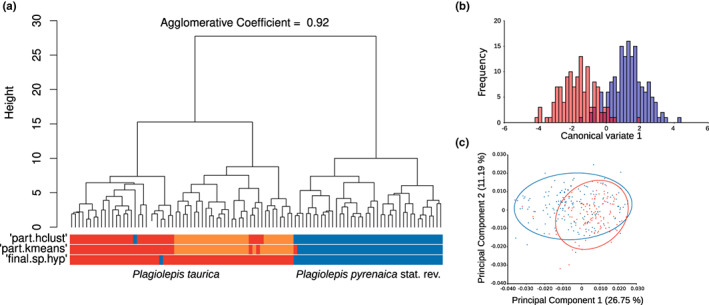
(a) Summary of analyses based on eight morphological characters obtained via traditional morphometrics (TM). The cladogram reflects distances inferred via NC‐Ward clustering. Bars below are results from the partitioning algorithm based on recursive thresholding (PART) using two clustering methods (“NC‐part.hclust and NC‐part.kmeans”). Final species hypotheses according to a LDA using majority decision of the three exploratory data analyses as hypotheses are shown is shown in a bar (“final.Sp.hyp”). (b) Canonical variate analysis based on morphological data obtained via geometric morphometrics (GM). The genetic species hypothesis has been used as a priori grouping information (blue, *Plagiolepis pyrenaica* stat. rev.; red, *Plagiolepis taurica*). (c) Principal component analysis based on morphometric data obtained from GM; blue dots, *P. pyrenaica* stat. rev.; red points, *P. taurica*, 95% equal‐frequency ellipses are correspondingly coloured

The final morphological species hypothesis based on TM data (characters defined in Table S1, measurements summarized in Table S2) showed a split into two groups, similar to the main split supported by the genetic data (Figures 1a and 3a). From the initially measured 21 characters, eight characters (CL/CW, PrOc, dTP, SL, Fu2, Fu3, PLG, BPDG) were retained for the final analysis after character selection by a stepwise LDA. The disagreement between classification of exploratory data analyses and final morphological species hypothesis was 1% in NC‐Ward, 1% in NC‐part.hclust, and 2% in NC‐part.kmeans (Figure 3a). The disagreement between the final genetic and the final morphometric species hypotheses was 8.7%. Imposing the RADseq‐SNP‐informed grouping into two main groups as hypothesis in a LDA considering 17 TM characters, morphometrics disagreed with the genetic hypothesis by 2.9%. Allocation of *P. pyrenaica* stat. rev. and *P. taurica* to the *P*. *pallescens* group was established by measuring the same characters (Appendix A, Table S3).

The Procrustes ANOVA revealed that the imaging error in the GM analyses was on average 12 times lower than the variation between any two measured specimens (*n* = 92, alpha = 0.05, *p* < .05). Thus, variation introduced via specimen positioning did not severely influence GM analyses. A PCA based on the Procrustes covariance matrix accounted for 38% of the observed variation when the first two axes were retained. However, PCA did not result in a clear separation of the data and provided no evidence to distinguish between the groups found via genetic analyses (Figure 3b). In a canonical variate analysis, to which the two‐cluster hypothesis derived from genetic data was passed as criterion, the GM data supported such a distinction (10,000 permutations, *p* < .05; Figure 3c).

### 3.4 Demographic modelling

**FIGURE 4:**
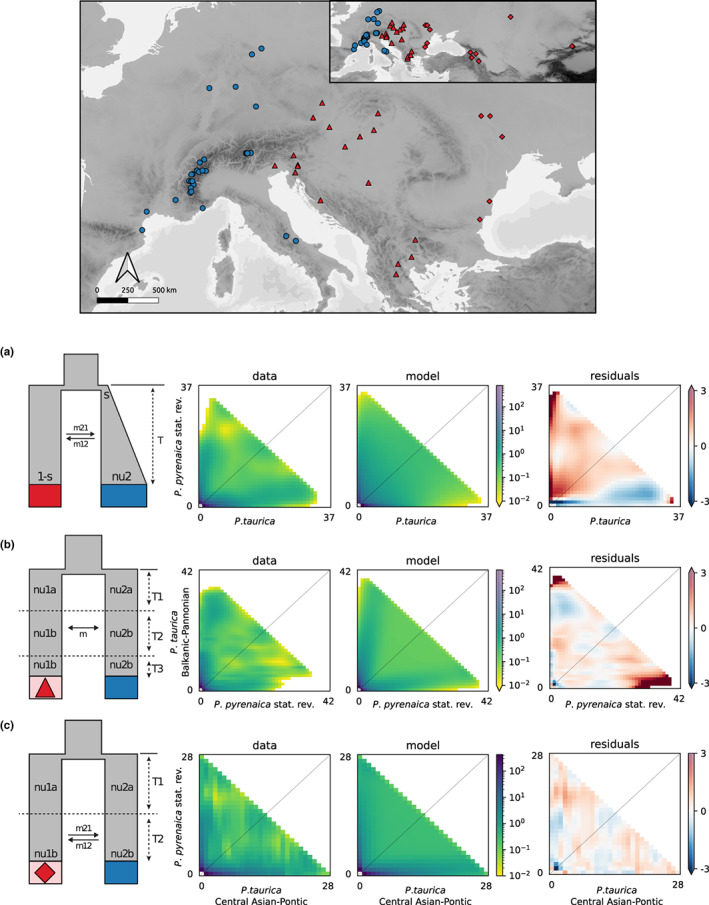
Map of populations used in demographic model selection and schematic visualization of the best performing demographic models for each pairwise comparison. For each comparison, the respective empirically inferred joint site frequency spectra (jSFS), the jSFS assumed by the model, and the corresponding residuals between empirical data and the model are given. (a) Localities of the included populations and their affiliation to the respective species hypotheses and subgroups (blue, *P. pyrenaica* stat. rev.; red, *P. taurica*; red triangles, *P. taurica* Balkanic‐Pannonian subgroup; red diamonds, *P. taurica* Central Asian‐Pontic subgroup). All model parameters are shown in Table 1. (b) Old founder event with exponential growth in first epoch, followed by continuous asymmetrical migration in second epoch (*P. taurica* and *P. pyrenaica* stat. rev.). (c) Three‐epoch model with size changes and periodical symmetric migration (P*. pyrenaica* stat. rev. and *P. taurica* Balkanic‐Pannonian subgroup). (d) Two‐epoch model with size changes and secondary contact (*P. pyrenaica* stat. rev. and *P. taurica* central Asian‐Pontic subgroup). The colour scales in jSFS plots represent the log‐scaled numbers of sites for each grid cell; numbers on *x*‐ and *y*‐axis depict the number of alleles

Model summaries and the corresponding AIC and ω_i_ values for all pairwise 2D model runs are given in Table S4. Parameter estimates for the best performing models are summarized in Table 1. Graphical summary of models and jSFS of empirical and model data, and residual plots are given in Figure 4. We refrained from transforming the obtained genetic units to biologically meaningful estimates as the main intent was to discern among diversification scenarios rather than obtaining exact parameter estimates.

**TABLE 1:** The best performing demographic models and the corresponding unscaled parameter estimates for three pairwise model comparisons (nu, effective population size; m, migration; T, time; s, founding fraction; for details see Portik et al., 2017, Charles et al., 2018). Parameters correspond to those shown in Figure 4

Populations	Model	Theta	nuA	nu1	nu2	nu1a	nu2a	nu1b	nu2b	m12	m21	m	T1	T2	T3	s
*P*. *pyrenaica* vs. *P*. *taurica*	founder_sec _contact_asym _two_epoch	211.23	0.683	0.431	6.017	‐	‐	‐	‐	1.664	0.547	‐	0.092	0.647	‐	0.113
*P*. *pyrenaica* vs. *P*. *taurica* (Balkanic‐Pannonian subgroup)	sec_contact_sym_mig _size_three_epoch	129.6	‐	‐	‐	6.61	0.49	13.92	8.19	‐	‐	1.66	0.63	0.10	0.03	‐
*P*. *pyrenaica* vs. *P*. *taurica* (Central‐Asian Pontic subgroup)	sec_contact _asym_mig	148	‐	‐	‐	0.39	22.62	8.63	2.21	0.44	0.49	‐	0.53	0.18	‐	‐

The best performing model (ωi = 1) for the pairwise comparison of *P*. *taurica* and *P*. *pyrenaica* stat. rev. was a two‐epoch founder event scenario with asymmetric migration in the second epoch (Figure 4a “founder_sec_contact_asym_two_epoch”, Záveská et al., 2021). The parameter describing the fraction of *P*. *taurica* that has been founding *P*. *pyrenaica* stat. rev. was small (*s* = 0.11). The first epoch with isolation and exponential growth was found to be shorter than the second epoch. For the second epoch, gene flow was suggested to have occurred asymmetrically and was three times larger from *P*. *pyrenaica* stat. rev. to *P*. *taurica* than vice versa.

The comparisons of *P*. *pyrenaica* stat. rev. and the subgroups of *P*. *taurica* resulted in models with periodical migration after initial post‐split isolation and population‐size changes. In both instances, the first epoch with isolation was longer than the second epoch with gene flow. Gene flow ratios in both cases were either symetrical or slightly asymetrical. More specifically, a three‐epoch model with symmetric migration in the mid‐epoch and population size changes performed best when comparing *P. pyrenaica* stat. rev. and *P. taurica* Balkanic‐Pannonian subgroup (ω_i_ = 1; Figure 4b “sec_contact_sym_mig_size_three_epoch”, Portik et al., 2017). This model also included population size increase in both populations after the first epoch, and a third, very short epoch of isolation. The best performing model for *P*. *pyrenaica* and *P*. *taurica* Central Asian‐Pontic subgroup comparison was a two‐epoch model with initial isolation and secondary contact and population size change (ω_i_ = 0.96; Figure 4c “sec_contact_asym_mig”, Portik et al., 2017). A population size increase was suggested for *P. taurica* Central Asian‐Pontic subgroup only, but not for *P*. *pyrenaica* stat. rev.

### 3.5 Ecological niche modelling

**FIGURE 5:**
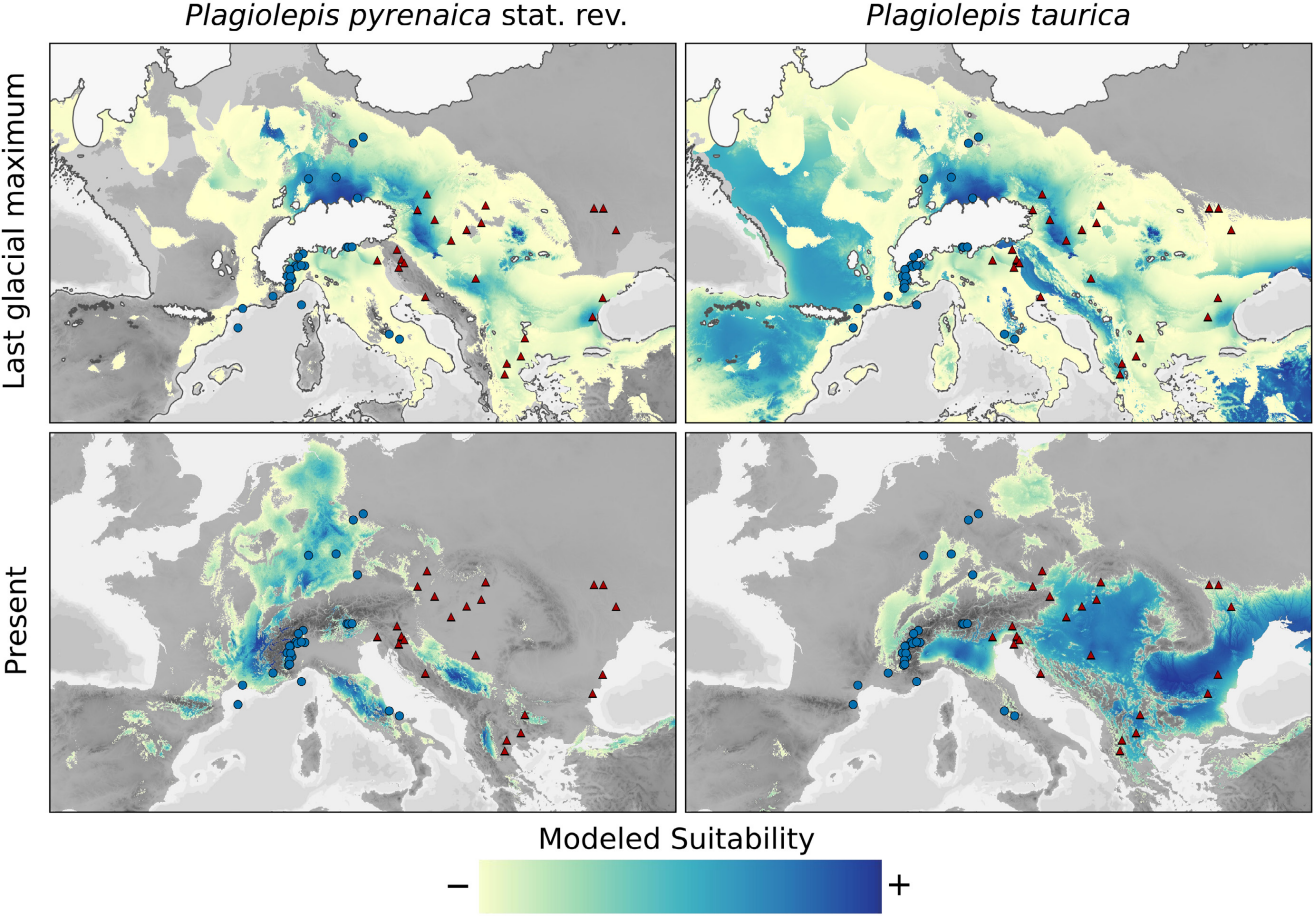
Modelled European distribution of *Plagiolepis pyrenaica* stat. rev. (circles) and *Plagiolepis taurica* (triangles) under current and last glacial maximum conditions (glaciated areas in white). Suitability is defined as the projected logistic probability of suitability above the species‐specific maximum training sensitivity plus specificity threshold

Model selection criteria suggested using linear‐quadratic‐hinge‐product features and a regularization parameter of 1 in the case of *P*. *pyrenaica* stat. rev. and linear‐quadratic features and a regularization parameter of three in the case of *P*. *taurica*. Predictive performance of both models was considered adequate (AUC *P*. *pyrenaica* stat. rev.: 0.96, AUC *P*. *taurica*: 0.85), and omission rates were close to zero (*P*. *pyrenaica* stat. rev.: 0.034, *P*. *taurica*: 0.043). The contribution of each climatic variable to the final model is summarized in Table S5. A comparison of both niches via a background test showed that the modelled niches of *P*. *pyrenaica* stat. rev. and *P*. *taurica* diverged significantly (Figure S7). A 10th percentile training present (P10) threshold was applied before visualization, and the final models are shown in Figure 5. Projection of the obtained models to LGM conditions showed a significant niche expansion for both *P*. *taurica* and *P*. *pyrenaica* stat. rev. (Figure 3b).

## 4 DISCUSSION

### 4.1 Species delimitation and taxonomic consequences

In this study, we reveal a large degree of diversification between the populations considered so far to represent a single nominal species, named *Plagiolepis taurica*. We find that Western and Central European *Plagiolepis* ants that have so far been assigned to the species *P*. *taurica* belong to *P*. *pyrenaica* Emery, 1921 stat. rev. In an extensive morphometric survey including all available type specimens of the *Plagiolepis pallescens* group (Appendix A), *Plagiolepis barbara* var. *pyrenaica* Emery, 1921 was identified as the correct name of the Central and Western European *Plagiolepis taurica* populations; the provisional name *Plagiolepis* OCCIDENTALIS that Seifert (2018) used on the basis of preliminary data corresponds with *Plagiolepis barbara* var. *pyrenaica*. The detailed morphometric survey of type material also showed that the synonymization of *P*. *taurica* with *P*. *pallescens* by Salata et al. (2018) is baseless and that *P*. *taurica* is a valid taxon (Appendix A, Tables S2 and S3).

Using evidence inferred from multiple disciplines (Figure 1) and following the gene and gene expression (GAGE) species concept (Seifert, 2020a), we thus reestablish *P. pyrenaica* stat. rev. as separate species. Phylogenetic analyses based on genomic SNPs and a mitochondrial gene congruently supported the divergence of *P*. *taurica* and *P*. *pyrenaica* stat. rev. (Figure 2b,c). A COI based phylogeny that included several *Plagiolepis* species showed that *P*. *taurica* and *P*. *pyrenaica* stat. rev. form a monophylum and that *P*. *pallescens* is their sister lineage, which is additional evidence against the synonymization of the two species with *P*. *pallescens* (Figure S5). Divergence between *P*. *taurica* and *P*. *pyrenaica* stat. rev. was further reflected by Bayesian clustering analyses that revealed admixture in populations from potential contact zones of *P*. *pyrenaica* stat. rev. and *P*. *taurica* (Figure 2a). However, explicit diversification models showed that the gene flow causing this pattern occurred only periodically or was an effect of secondary contact between two well separated evolutionary entities (Figure 4, Table 1).

Naming the Central and Western European populations *P*. *pyrenaica* stat. rev. was confirmed by NC‐clustering analyses based on TM data (Figure 3). These data supported a split into two clusters over a split into three distinct clusters (as partly suggested by genomic data; Figure 3). Some discordance of morphological and genetic species hypotheses was found in Slovenia (three individuals) and Croatia (two individuals). These specimens were sampled from a contact zone of *P*. *pyrenaica* stat. rev. and *P*. *taurica* where also admixture was detected (Figure 2a). However, in accordance with the GAGE species concept, classification based on thousands of genomic SNPs is to be preferred over hypotheses based on 17 morphological characters. GM data failed to reproduce a two‐cluster grouping when they were analysed by exploratory data analysis (Figure 3b). Yet, both the TM and GM data fully confirmed the genetically defined clusters when run in a linear discriminant analysis with hypotheses proposed by genetic classification (Figure 3c). While GM of ant heads has shown to be suitable to differentiate between closely related evolutionary units in ant genera with a strongly structured exoskeleton such as *Myrmica* (Bagherian Yazdi et al., 2012), this was not the case for *Plagiolepis*. As we can rule out significant measurement errors, our data showed no significant bias of this sort, we conclude that GM of *Plagiolepis* head capsules does not yield sufficient information to differentiate between closely related entities.

Genetic data did not reveal substructure within *P*. *pyrenaica* stat. rev. (Figure 2a,b,d, Figure S6), which we interpret as evidence for the genetic integrity of the species and the lack of potential introgression from other *Plagiolepis* species. In contrast, distinct substructure and also incongruent tree topologies between SNP and COI based trees were found within *P*. *taurica* (Figure 2, Figure S6). Phylogenetic analyses and Bayesian cluster analyses grouped populations that were closest to the type locality of *P*. *vindobonensis* to a well‐supported Balkanic‐Pannonian subgroup and those of *P*. *taurica* to a well‐supported Central Asian‐Pontic subgroup (Figure 2a, Figure S6). While incongruent topologies (Figure 2b,c) might hint at a more complex history of hybridization between these two subgroups, we will not interpret this further as we cannot rule out that this finding only reflects limited marker coverage of the used COI fragment. However, we found that the SNP based genetic and spatial divergence between these clusters reflect the taxonomic dispute that arises when considering *P*. *taurica* as a senior synonym of *P*. *vindobonensis*. TM data suggested a similar divergence pattern that was, however, not supported significantly (Figure 3a, Supporting Information Data S1). In accordance with the GAGE species concept, we therefore refrain from reviving *P*. *vindobonensis* from synonymy and suggest to follow the classification of Seifert (2018).

### 4.2 Speciation mode

We suggest a speciation scenario according to which *P*. *pyrenaica* stat. rev. and *P*. *taurica* have split via peripatric speciation. This implies that the speciating evolutionary unit (here *P*. *pyrenaica* stat. rev.) initially separated from the peripheral range of a central evolutionary unit (here *P*. *taurica*) as a consequence of a range contraction. By employing an explicit test strategy comparing different evolutionary scenarios, we find that the most likely model fits a peripatric speciation hypothesis (i.e., a model including an old founder event followed by a period of isolation with exponential growth of *P*. *pyrenaica* stat. rev. after initial split of a small fraction of *P*. *taurica*, Figure 4a, Table 1). A similar peripatric speciation scenario was suggested for steppe ants from the genus *Messor* (Steiner et al., 2018), which could, however, not be substantiated by direct evidence from more rigorous tests due to a lack of data. Given the presented evidence, we reject the hypotheses of allopatric speciation and of parapatric speciation as outlined in the introduction.

Populations founding *P*. *pyrenaica* stat. rev. were most probably separated from *P*. *taurica* as a consequence of a steppe‐range contraction. Eurasian steppes contracted and expanded repeatedly throughout the Pleistocene, driven by the interplay of warm and cold stages (Hurka et al., 2019; Sadori et al., 2016). The initial split between the two species was dated to have occurred in the Pleistocene 1.5 to 1.33 million years ago (Ma) (median age of 1.5 Ma based on a single gene COI based phylogeny, Kirschner et al., 2020; median age of 1.33 Ma based on genomic data, Kirschner et al., 2022). This slightly precedes a period known as mid‐Pleistocene transition (MTP) in which glaciation cycles became significantly longer while warm stages became shorter (Willeit et al., 2019). This probably promoted an unprecedented expansion of Pleistocene steppes and their biota in cold stages (Kirschner et al., 2022). We speculate that events of the MTP facilitated the separation of the two ant species by enabling populations ancestral to *P*. *pyrenaica* stat. rev. to establish long‐term stable populations that were able to endure warm stage steppe contractions in Western and Central Europe.

The here presented ENM suggested similar dynamics for the LGM and for present‐day conditions, that is, a cold‐stage (LGM) niche expansion and a warm‐stage (present day) contraction for *P*. *taurica* and *P*. *pyrenaica* stat. rev. (Figure 5). These niche models need to be interpreted with some care: All models were exclusively informed by climatic data, whereas other parameters that have been shown to be important for inferring ecological niches for Palearctic ants, such as soil type, inclination, and exposition, could not be incorporated on the modelled scale (Seifert, 2017). In other words, suitable areas in the models reflect a climatic niche that will be occupied by the species if steppic grassland is present. In the temperate forest climate of Europe, this is the case if edaphic conditions hinder forest establishment. For this reason, the present‐day niche models are probably overpredictions especially in the case of *P*. *pyrenaica* stat. rev. that, in its present‐day, warm‐stage distribution, exclusively occurs in steppe habitats that are embedded in a matrix of temperate forests. However, given this, we also stress that potential habitats within the modelled niches in cold stage conditions were also much more frequent as trees were definitely absent from large parts of the modelled niche at this time, and steppe grasslands were the dominant vegetation type (Binney et al., 2017). Finally, we also interpret the multiepoch character of demographic models, and the respective population‐size changes as consequences of the steppe‐range dynamics as outlined above (Figure 4b,c).

In the emphasized mode of peripatric speciation, diverged peripheral lineages are often reincorporated by the re‐expanding central evolutionary unit. However, this only applies if no reproductive barrier of some sort has arisen in phases of allopatric isolation – which in case of *P*. *pyrenaica* stat. rev. and *P*. *taurica* is not obvious. However, the limited dispersal capacity of small ants such as *Plagiolepis* (Helms, 2018) might have played a central role here. Limited dispersal ability probably favours separation during warm stages despite geographic proximity and thus also impeded the central evolutionary entity (*P*. *taurica*) from overrunning the speciating one (*P*. *pyrenaica* stat. rev.) during cold stage steppe expansions. While small body size promotes passive long‐distance dispersal as “air plankton”, it similarly allows active flight or ground movements over short distances only (Cardoso et al., 2015; Dudley, 2000; Stevens et al., 2014; Ströher et al., 2019). Colonization of emerging habitats in both cold stages (emerging steppes all over Europe) and warm stages (previously ice‐covered Alpine steppes) was probably slow and probably followed a “leading edge” scenario (e.g., Hewitt, 2008, reviewed in Waters et al., 2013). Newly emerging, unoccupied niches probably were constantly monopolized on a local and regional level by the local refugial populations via short‐distance dispersal, while the odd and infrequent incoming long‐distance dispersers probably were assimilated by more frequent and established populations. The detected admixture, however, might hint at where the respective leading edges of both species have had contact previously (Figure 2a).

We did not find evidence for significant range overlaps of the present‐day distributions of *P. pyrenaica* stat. rev. and *P*. *taurica*, while large‐scale range overlaps of *P*. *taurica* and *P*. *pyrenaica* were modelled for the LGP (Figure 5). Assuming that the present‐day, warm‐stage distributions are at least partly analogous to those in previous warm stages, *P*. *taurica* and *P*. *pyrenaica* stat. rev. must have been repeatedly separated during Pleistocene warm stages. A large number of admixed populations on the Western and Southern Balkan and the Pannonian Basin (Figure 2a) and incongruence in the morphometrics‐informed clustering hint at a potential contact zone in this region.

In line with the initially formulated hypothesis, demographic models comparing *P*. *pyrenaica* stat. rev. and the two subgroups of *P*. *taurica* suggested that gene flow between *P*. *taurica* and *P*. *pyrenaica* stat. rev. was not continuous and occurred after phases of clear allopatry (Figure 4b,c, Table 1). Specifically, migration was either symmetric and restricted to a certain epoch in case of *P*. *pyrenaica* stat. rev. and the Balkanic‐Pannonian subgroup (Figure 4b) or asymmetric from the Central Asian‐Pontic subgroup to *P*. *pyrenaica* stat. rev. in a phase of secondary contact (Figure 4c). We interpret this periodical gene flow as evidence of contact between P. *t* *au* *rica an*d *P*. *pyrenaica* stat. rev. in the periods of steppe expansions, that is, the LGP. The symmetric gene flow found between *P*. *pyrenaica st*at. rev. and the Balkanic‐Pannonian subgroup of *P*. *tauica* further shows that the admixture signal on the Balkan and in the Pannonian basin results not only from P. *taurica* expanding westwards but also from an eastward expansion of *P*. *pyrenaica* stat. rev. as suggested by ENM (Figure 5) and visible in the Bayesian clustering (Figure 2).

Local adaptation can be another important factor in facilitating and/or maintaining separation between speciating entities (Schluter, 2001). While we do not have direct evidence of such processes, we found that the modelled niches of both species were significantly divergent (Figure 5, Table S5, Figure S7) and interpret this as indirect evidence for ecological speciation (McCormack et al., 2010). Data from Seifert (2018) that indicate an allochronic differentiation between *P*. *taurica* and *P*. *pyrenaica* stat. rev. point in a similar direction. These data show that the temporal occurrence of alates differs between the two species: alates appear, on average, four weeks earlier in *P*. *pyrenaica* stat. rev. than in *P*. *taurica* (one‐way ANOVA *F* = 8.644, alpha = 0.05, *p* =.009, *n* = 20; data from Seifert, 2018). A spatially and temporally more evenly sampled data set would allow a more robust conclusion here. However, we stress that species‐specific timing of alate appearance hints at a species‐specific adaptation to the significantly different climatic niches (Figure S7).

Another factor that is known to propagate speciation in arthropod lineages is reproductive incompatibility caused by infection with endosymbiotic bacteria such as *Wolbachia* (Rokas, 2000). While such infections are common in ants (*Wolbachia* infects over 34% of all ant species), related cospeciation has been rarely addressed (but see, e.g., Steiner et al., 2018) and has been suggested to be rare (Russell et al., 2012, 2017). Nevertheless, the role of endosymbionts on lineage diversification over large geographic and temporal scales, such as in the framework of this study, might be an intriguing topic for future studies.

In combining the available evidence, we make plausible that the steppe expansions and contractions in the Pleistocene were important drivers of diversification and speciation in the species pair of *P*. *pyrenaica* stat. rev. and *P*. *taurica*. While the mode of speciation has not been explicitly studied in such detail for any steppe species, the here revealed separation of ancestral lineages in Eastern Europe and Western Asia (*P*. *taurica*) versus derived lineages in Central and Western Europe (*P*. *pyrenaica* stat. rev.) is in line with scenarios emphasized for Eurasian steppe biota in general (Kirschner et al., 2020). Given the dynamics of steppe expansions and contractions, we emphasize that the elaborated peripatric speciation scenario, that is, Western and Central European lineages of Eurasian steppe biota evolving in peripatry since as long as the mid‐Pleistocene, could be characteristic of European steppe biota in general. We want to highlight that the here presented explicit speciation scenario is now available for being tested in other potential candidate systems. In this line, we also want to point out that the potentially overlooked lineage diversity in Eurasian steppe biota might be reflected by widespread morphological crypsis, especially in understudied groups such as arthropods. As Eurasian steppe‐range dynamics have not yet been understood in detail and given the high conservation value of steppes (Török et al., 2016; Kirschner et al., 2020), we also highlight the need of additional case studies targeting typical steppe biota that focus on taxonomy.

## AUTHOR CONTRIBUTIONS

Philipp Kirschner, Birgit C. Schlick‐Steiner, and Florian M. Steiner conceived the project and designed the study and collected specimens in the field. Philipp Kirschner did RADseq‐related laboratory work and analysed RADseq data. Joelle Kröll generated GM data. Bernhard Seifert generated TM data and performed all TM and GM analyses and revealed the taxonomic background by investigation of type specimens. Philipp Kirschner performed niche modelling. Philipp Kirschner, Birgit C. Schlick‐Steiner, Bernhard Seifert, and Florian M. Steiner cowrote the manuscript. Joelle Kröll and members of The STEPPE Consortium contributed to the manuscript writing, provided expertise in the laboratory and for data analyses, and collected in the field.

## 5 CONFLICT OF INTEREST

The authors declare no conflicts of interest.

## THE STEPPE CONSORTIUM MEMBERS

The STEPPE (Steppe Taxa in Eurasia: Phylogeny, Phylogeography, and Ecology) Consortium consists of (alphabetic order):

Wolfgang Arthofer (Department of Ecology, University of Innsbruck, Austria)

Božo Frajman (Department of Botany, University of Innsbruck, Austria)

Alexander Gamisch (Department of Biosciences, University of Salzburg, Austria)

Andreas Hilpold (Institute for Alpine Environment, Eurac Research Bolzano/Bozen, Italy)

Philipp Kirschner (Department of Ecology, University of Innsbruck, Austria)

Ovidiu Paun (Department of Botany and Biodiversity Research, University of Vienna, Austria)

Isabel Sanmartín (Real Jardín Botánico, CSIC, Madrid, Spain)

Birgit C. Schlick‐Steiner (Department of Ecology, University of Innsbruck, Austria)

Peter Schönswetter (Department of Botany, University of Innsbruck, Austria),

Florian M. Steiner (Department of Ecology, University of Innsbruck, Austria)

Emiliano Trucchi (Department of Life and Environmental Sciences, Marche Polytechnic University, Via Brecce Bianche, 60131, Ancona, Italy)

Eliška Záveská (Department of Botany, University of Innsbruck, Austria)

## Supporting information


Data S1.

## Data Availability

RADseq raw reads have been made available via NCBI's Short Read Archive (accession numbers given in Supporting Information Data S1). Mitochondrial DNA sequences have been deposited on Genbank (accession numbers given in Supporting Information Data S1). Collection data for each specimen are presented in tabular format (Supporting Information Data S1). Morphometric raw data have been deposited in a dryad library 10.5061/dryad.c866t1g9f.

## APPENDIX 1 Taxonomic position of *Plagiolepis pyrenaica* stat. rev. and *Plagiolepis taurica* within the Westpalaearctic *Plagiolepis* species

Following Seifert (2020b) and based on investigation of type specimens (measured characters and abbreviations are defined in Table S1) and, in some cases, only of their images in AntWeb.org, the independent species of the genus *Plagiolepis* of the Westpalaearctic can be subdivided in three major groups:

- the *Plagiolepis pallescens* complex which is characterized by widely spaced basal pits of pubescence hairs on dorsum of first gaster tergite and the fourth funiculus segment not being much longer than the third. The data of 321 workers of at least five good species are BPdD 30.17 ± 2.07 μm and F4/F3 1.158 ± 0.057. Mean BPdG translates into 1099 pubescence hairs/mm^2^. Without making implications here on their potential species status by use of binary names, the described taxa of this complex are *P. pallescens* Forel, 1889; *P. minu* Forel, 1911; *P. taurica* Santschi, 1920; *P. sordida* Santschi, 1920; *P. ancyrensis* Santschi, 1920; *P. vindobonensis* Lomnicki, 1925; *P. compressa* Radchenko, 1996; *P. dlusskyi* Radchenko, 1996; *P. calva* Radchenko, 1996; *P. pyrenaica* Emery, 1921. In a separate section below we consider the taxonomic status of all these taxa.
- the taxa close to *P. pygmaea* Latreille, 1798 which are characterized by rather widely spaced basal pits of pubescence hairs on dorsum of first gaster tergite and by the fourth funiculus segment being much longer than the third. The data of 35 workers are BPdD 24.82 ± 2.82 μm and F4/F3 1.596 ± 0.078. Mean BPdG translates into 1623 pubescence hairs/mm^2^. Without making implications on their potential species status by using here binary names, the described taxa of this group are *P. pygmaea* Latreille 1798, *P. obscuriscapus* Santschi 1922 and *P. karawajew*i Radchenko, 1989.
- the *Plagiolepis schmitzii* complex which is characterized by narrowly spaced basal pits of pubescence hairs on dorsum of first gaster tergite and the fourth funiculus segment not being much longer than the third. The data of 109 workers of probably three good species are BPdD 16.53 ± 1.98 μm and F4/F3 1.189 ± 0.056. Mean BPdG translates into 3660 pubescence hairs/mm^2^. Without making implications on their potential species status by using binary names, the described taxa of this group are *P. schmitzii* Forel, 1895; *P. barbara* Santschi, 1911; *P. atlantis* Santschi, 1920; *P. crosi* Santschi, 1920; *P. kabyla* Santschi, 1920; *P. maura* Santschi, 1920; *P. polygyna* Santschi, 1922 and *P. tingitana* Santschi, 1936; *P. perperamus* Salata et al., 2018; *P. invadens* Seifert, 2020b. In all taxa type specimens were subject to morphometric investigation (Seifert, 2020b).

### *Plagiolepis pyrenaica* Emery, 1921 stat. rev.

**Type material**: Investigated were two syntype workers from MCSN Genova labelled “France Pyr. occ. Banyuls”, “SYNTYPUS *Plagiolepis barbara* var. pyrenaica Emery, 1921”, and “ANTWEB CASENT0905139”.

**All material examined**: A total of 36 nest samples with 105 workers were subject to morphological investigation. For details see Supporting Information Data S1.

**Geographic range**: According to the 36 samples treated in this paper, additional samples investigated only morphometrically by B.S., and literature reports with a clear geographic argument found on the Channel Islands, in France, S Belgium, Germany north to 51.7°N, Switzerland, Italy and W Slovenia. At the southern slope of the Alps at 45.8°N ascending to 1370 m.

**Diagnosis of worker**: (Table S2, Figures S8 and S9)

Very small, mean CS 463 μm. Head slightly more elongated than in *P. taurica* (mean CL/CW_450_ 1.133 vs. 1.111). Distance of anterior tentorial pits larger than in *P. taurica* (mean dTP/CS_450_ 0.520 vs. 0.508). Eye medium‐sized in terms of the *P. pallescens* group (mean EL/CS_450_ 0.282). Preocular distance larger than in *P. taurica* (mean PrOc/CS_450_ 0.235 vs. 0.225). Postocular distance medium‐ sized in terms of the *P. pallescens* group (mean PoOc/CL_450_ 0.369). Scape significantly longer than in *P. taurica* (mean SL/CS_450_ 0.935 vs. 0.911). Third segment of antennal funiculus longer than in *P. taurica* (mean F3/CS_450_ 9.13 vs. 8.61%). Length ratio of fourth versus third funiculus segment small in terms of the genus *Plagiolepis* (F4/F3_450_ 1.129). Mesosoma longer than in *P. taurica* (mean ML/CS_450_ 1.184 vs. 1.146). Pubescence on whole body sparse and more or less decumbent; length of pubescence hairs on posterior dorsum of first gaster tergite longer than in *P. taurica* (mean PLF/CS_450_ 10.00 vs. 9.34%) and longest within the whole *P. pallescens* group. Mean density of pubescence hairs on posterior dorsum of first gaster tergite only 1090/ mm^2^, the distance of their basal pits as a result widely spaced (BPdG_450_ 30.3 μm). Smooth, pointed and erect setae of 65–100 μm length are present near to anterior and posterior clypeal margin and near to posterior margin of gaster segments. Few shorter, slightly pinnate and erect setae are present on posterior vertex and occasionally on posterior pronotum. Cuticular surface of all body parts glabrous, very shining and with rare, scattered elements of stickman‐like microsculpture. Head, mesosoma, petiole, gaster and usually antennal funiculus dark to blackish brown; scape, mandibles, coxae and legs usually paler yellowish brown to yellowish. Yellow colour morphs as they occur in some species of the *P. pallescens* and *P. schmitzii* group are unknown in *P. pyrenaica* stat.nov. and *P. taurica*.

**Biology**: It is found in very xerothermous grassland, more rarely in xerothermous woodland or xeric alluvial biotopes (“Heißländen”). In its German distribution, urban habitats are avoided but the species may be temporarily introduced there with plant or soil material and may even occur in buildings. In its Alpine distribution, the species occurrs only in steppic grassland of the inner‐Alpine dry valleys, with exception of Valais (Switzerland) exclusively south of the Alpine main ridge (Eastern Alps: only present in Vinschgau and upper Etsch and Eisack valleys; Western Alps: e.g., Val d‘Aosta, Val Susa, Val Durance, Val Tarentaise). In the Apennine the species is present in continentally influenced, dry grasslands of the Central Apennine, and seems to be mostly replaced by *P*. *pygmaea* in more submediterranean grassland habitats (personal observation Kirschner). In France apart, from the Alps, and on the Iberian peninsula the species seems to prefer xeric grasslands, but also occurrs in sandy habitats (dunes at Saint Cyprien Plage, France) and grassland habitats under more submediterranean influence. In Germany known from 47 outdoor sites. Population densities near the northern distributional border are low: mean and maximum density on three study plots in Sachsen‐Anhalt 6.8 and 10 nests/100 m^2^. Nests are under stones, in rock crevices, or in soil. The colonies are polygynous, nests often contain >20,000 workers, spots with dense clusters of nests indicate polydomy. According to Thurin et al. (2011), relatedness within colonies of a population from S. France is high because of sib‐mating and a high average relatedness of the male mates of a queen (fixation index Fit = 0.25). Occurrence of alates 15 June ± 15 d (14 May, 9 July) *n* = 11. This is 1 month earlier than in *P. taurica* (12 July ± 25 days, 14 June, 23 August *n* = 9). Queens are highly polyandrous, their effective mating number ranges between 3 and 11. Colony foundation occurs frequently by adoption in or near to the home colony, independent single queen colony foundation is also possible. Nutrition is strongly nectarivorous and trophobiotic – also with caterpillars of Lycaenidae. May form very busy trails to long‐term food sources.

**Comments**: The phenotypes of *Plagiolepis taurica* and *P. pyrenaica* stat. rev. are extremely similar. In the most separating morphometric character SL/CS, 66% of individuals are placed within the interspecific overlap range described by the 95% confidence intervals. Therefore, a simple means for the morphological discrimination of *P. pyrenaica* stat. rev. and *P. taurica* cannot be given.

### Further synonymies within the *Plagiolepis pallescens* complex

The taxa of the *P. pygmaea* and *schmitzii* complex are morphologically well separated from those of the *P. pallescens* group and are not discussed here. However, assessing a large number of populations that were previously assigned to *P. taurica*, we had to consider synonymies within the *P. pallescens* group.

*P. pallescens*, as the oldest taxon of the group, differs from both *P. pyrenaica* stat. rev. and *P. taurica* by a much longer scape and fourth funiculus segment (Table S2) and can be fully separated from the two by a PCA. Heterospecificity of *P. pyrenaica* stat. rev. from *P. pallescens* is further supported by a separate zoogeography. The separation of *P. pallescens* from *P. tauric*a is presented here in more detail because they show zoogeographic overlap in the Balkans at least and because Salata et al. (2018), simply comparing photos in AntWeb.org, assumed the former to be a senior synonym of the latter. Using the RAV‐corrected characters SL/CS_450_, PLG/CS_450_, dTP/CS_450_, Proc/CS_450_ and F3/CS_450_, the first factor of a PCA is 3.000 ± 1.009 [1.620, 4.038] in 12 workers of *P. pallescens* and –0.186 ± 0.640 [–1.669, 1.496] in 194 workers of *P. taurica* (ANOVA, *F*_1,204_ = 259.22, *p* << .0001). Running a PCA considering CS and all 16 RAV‐corrected characters and using the first three components of this PCA in a LDA, all type specimens of *P. pallescens* and *P. ancyrensis* are allocated with *p* = 1.000 to the *pallescens* cluster, whereas the types of *P. taurica* and examples from near the type locality of *P. vindobonensis* in Vienna are allocated to the *taurica* cluster with both *p* = 1.000.

The next oldest taxon, *P. minu*, known from W Anatolia in the types and another sample, differs from both *P. pyrenaica* stat. rev. and *P. taurica* by smaller eyes, larger pre‐ and post ocular distance and shorter gaster pubescence (Table S3).

*P. sordida*, known so far only by the type sample from Tunisia and having been described with *P. taurica* in the same paper, is similar to the latter (Table S2). Yet, a synonymy appears unlikely due to separate zoogeography and full separation in a PCA considering the characters EL/CS_450_, PoOc/CL_450_, and BPdG_450_. If turning out as synonymous by future investigations, the name *P. taurica* should be given priority by revisors decision. The separation of *P. sordida* from *P. pyrenaica* stat. rev. is strongly supported by a PCA and zoogeography.

The taxa *P. compressa*, *P. dlusskyi* and *P. calva*, all described by Radchenko in 1996, do not pose a risk of synonymy with *P. pyrenaica* stat. rev. alone by the widely separate zoogeography. The type series of *P. compressa* plus a sympatric sample from Kopet Dagh are placed widely separate from *P. pyrenaica* stat. rev. in a PCA and but close to *P. taurica*. *P. compressa* is considered here as a deviating regional population of *P. taurica* with a scape length at the upper end of the *P. taurica* range of variation. *P. dlusskyi* from Armenia and *P. calva* from Turkmenistan, in which only pictures of type specimens in AntWeb.org could be studied, are synonymized here with *taurica* as long as a data‐based argumentation for heterospecificity is lacking.

As conclusion from all these findings, there is no described taxon that could turn out as senior synonym of both *P. pyrenaica* stat. rev. and *P. taurica*. Below we list the six recognized species of the *P. pallescens* group with synonymies and comments.

### *Plagiolepis pallescens* Forel 1889

*Plagiolepis pygmaea pallescens* Forel, 1889 [type investigation]

Terra typica: Islands of Rhodos and Kapathos/Greece. Investigated were two type workers labelled “P. pygmaea Ltr …Rhodos ….Karpatho v. Oertzen” [poorly legible], “v. pallescens For.”, “Typus”, “ANTWEB CASENT 09009854”. The lectotype designation by Salata et al. (2018) was performed by inspection of a photo (ANTWEB CASENT 09009854) and not by direct investigation of specimens. Unfortunately, just the photographed specimen, situated on the second cardboad of the pin, has a crushed head. The undamaged lower specimen is herewith designated as lectotype.

*Plagiolepis maura* var. *ancyrensis* Santschi, 1920 [syn. by type investigation]

Type locality: Angora / Turkey. Investigated were two gynes two type workers from NHM Basel, labelled “Plagiolepis maura v ancyrensis Sants”, “Asie Min. Angora (G.d.Kerville), “type”, “ANTWEB CASENT 0912415 at TOP”, “Lectotype (at top) *Plagiolepis ancyrensis* Santschi des. Seifert, 2018” (fortunately the same specimen as published but not labelled by Salata et al., 2018). The specimens were washed off and remounted by B. Seifert in January 2018. The lectotype is by NUMOBAT data excellently fitting to the lectotype of *P. pallescens* but the saturated amber yellow colour of the latter poses questions. The second worker and the two gynes in the type series belong to *Plagiolepis atlantis* Santschi, 1920.

### *Plagiolepis minu* Forel, 1911

*Plagiolepis pygmaea ssp. minu* Forel, 1911 [type investigation]

Locus typicus: Ayvalik near Smyrna. Investigated were three syntype workers from MNH Genéve

labelled “Typus”, “P. pygmaea v. minu Forel type …Aivaly p. Smyrne… bois (Forel)”, “ANTWEB CASENT0909856”. A synonymy with *P. taurica* is unlikely because of smaller eyes, larger pre‐ and postocular distance and shorter gaster pubescence (Table S2). The first factor of a PCA run with PrOc/CS_450_, PoOc/CL_450_, EL/CS_450_, PLG/CS_450_ and Fu4/Fu3_450_ is −0.080 ± 0.874 (–2.493, 2.396) in 194 workers of *P. taurica* and 3.121 ± 0404 (2.883, 3.838) in five workers of *P. minu*.

### *Plagiolepis taurica* Santschi, 1920

*Plagiolepis maura* var. *taurica* Santschi, 1920 [type investigation]

Locus typicus: Crimea /Ukraine. Investigated were 3 syntype workers in NHM Basel labelled “P. maura Sant v taurica type Sant”, “Crimee Stary …Teodosie Mejunof”, “ANTWEB CASENT 0912433 middle”. The specimens were washed off and remounted by B. Seifert in January 2018, CASENT 0912433 is the second specimen on the pin.

*Plagiolepis vindobonensis* Lomnicki, 1925 [syn. by zoogeography and topotypical samples]

Locus typicus: Wien (Sievering) 2.6. 1915

*Plagiolepis compressa* Radchenko, 1996 [syn. by type investigation]

Originally described as *Plagiolepis compressus* Radchenko, 1996

Locus typicus: Kopet Dagh /Turkmenistan. Investigated were three paratype workers labelled “Ipai‐Kala, kserofitnaya, dol. sozlakami, 29.V.71, 71‐115, Dlussky”, CASENT 0917578.

*Plagiolepis calva* Radchenko, 1996 [syn. by photo inspection]

Originally described as *Plagiolepis calvus* Radchenko, 1996

Locus typicus: Kopet Dagh /Turkmenistan. According to AntWeb.org CASENT 0917579 types labelled “Ipai‐Kala, Kopet Dagh, 24. V. 71 Dlussky, 71‐78”. Synonymized as long as a data‐based argumentation for heterospecificity is lacking.

*Plagiolepis dlusskyi* Radchenko, 1996 [syn. by photo inspection]

Locus typicus: Armenia. According to AntWeb.org CASENT 0917484 labelled as “Karabkhlar, Vedi, Armen. n II 28, Dlussky 17. VI. 960”. Synonymized as long as a data‐based argumentation for heterospecificity is lacking.

### *Plagiolepis sordida* Santschi, 1920

*Plagiolepis maura sordida* Santschi, 1920 [type investigation]

Locus typicus: Tunisia. Investigated were four type workers from NHM Basel labelled “Tunisie La Quareb Santschi”. Only the type sample is known. It is treated here as good species because a synonymy with *P. taurica* is unlikely due to geography and a PCA of _RAV‐corrected_ EL/CS_450_, PoOc/CS_450_, BPdG_450_ (Table S3).

### *Plagiolepis isis* Santschi, 1938

*Plagiolepis pallescens* var. *isis* Santschi, 1938 [type investigation]

Locus typicus: Egypt. Investigated were two type workers from NHM Basel labelled “Plagiolepis pallescens For. v. isis Sant”, “EGYPT Min. Agric. (Egypt) Coll. A. Alfieri”, “NEST IN STEM OF PHRAGMITES WEED GHEZIREH 23.8.23”, “Type”, “ANTWEB CASENT 0912422”. It is a good species characterized by a very frontal eye position (PrOc/PoOc 0.528), small eyes, a very high ratio SL/F2 of 11.07 and pale yellowish colour. The type locality is most probably Geziret el‐Hagar (30.558°N, 30.838°E, 14 m). A synonymy with *taurica* is unlikely due to geography, colour and large F2/CS_450_.
