# Supplementary material for: Phylogenomic inference and demographic model selection suggest peripatric separation of the cryptic steppe ant species Plagiolepis pyrenaica stat. rev
Source: Mol Ecol. 2023 Jan 20;32(5):1149–68. doi: 10.1111/mec.16828 (PMC10946478; doi:10.1111/mec.16828)
Supplement: Supplementary file 1 — Data S1. [file MEC-32-1149-s001.zip › MEC_16828_Supplementary_Material_Kirschneretal.docx]

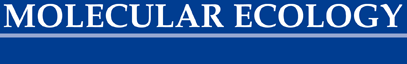


Supplemental Information for:

**Phylogenomic inference and demographic model selection suggest peripatric separation of**

**the cryptic steppe ant species *P*. *taurica* and *P*. *pyrenaica* stat. rev.**

# Philipp Kirschner, Bernhard Seifert, Joelle Kröll, the STEPPE Consortium, Birgit C. Schlick-Steiner & Florian M. Steiner

Corresponding author: Philipp Kirschner, philipp.kirschner@gmail.com

Supplementary Table 1. Definition of morphological features used for traditional morphometrics (TM).

| **Character** | **Definition** |
| --- | --- |
| **BPdG** | Mean distance between the base points of pubescence hairs on dorsal plane of 1 st gaster tergite. Not based on direct measurement but calculated from the sqPDG and PLG data. |
| **CL** | Maximum head (cephalic) length in median line; the head must be carefully tilted to the position with the true maximum. Excavations of hind vertex and/or clypeus reduce CL. |
| **CS** | Cephalic size; the arithmetic mean of CL and CW, used as a less variable indicator of body size. |
| **CW** | Maximum measurable head (cephalic) width. The position of measuring line is defined alone byt he maximum and may across or behind the eyes, varying between the genera considered. |
| **dAN** | Minimum distance of the inner (centripetal) margins of antennal socket rings which is best measurable in dorsofrontal view (Fig. 271). |
| **dFov** | Mean distance between foveolae on cuticular surface. Count the number of punctures N found within a total area A; dFov = square root (A/N). |
| **dTP** | Distance of the centres of clypeal tentorial pits. |
| **EL** | Large diameter of the elliptic compound eye measured over all structurally visible ommatidiae –i.e., also including unpigmented ones in a marginal position. |
| **FuN** | Median length of funiculus segment N = (here 1-3) in dorsal view. Dorsal view is given when the swiveling plane of 1st funiculus segment is positioned in the visual plane. Take care to really measure median length (the segment‘s sides may have unequal lengths!) and to recognize the real distal margin of the segment. The latter may have a very thin cuticle, frequently producing a narrow, shining ribbon that seems to be, by optical impression, demarcated from the rest of the segment. |
| **ML** | Mesosoma length without neck shield (fringe), posterior measuring point: caudalmost point of metapleuron; parallelity of the measuring line to the longitudinal mesosomal axis has to be considered – i.e, in lateral view, the anterior measuring point is found at a lower level of focus. If one of the measuring points is concealed: the distance of anterior measuring point from posteriormost margin of pronotum is 51.0% of ML. |
| **MW** | Maximum mesosoma width; this is in workers pronotal width, in gynes it is measured anteriorly of the tegulae. |
| **PLG** | Mean length of at least 7 pubescence hairs on dorsal plane of 1st gaster tergite. |
| **PoOc** | Postocular distance. Use a cross-scaled ocular micrometer and adjust the head to the measuring position of CL. Caudal measuring point: median occipital margin; frontal measuring point: median head at the level of the posterior eye margin. Note that many heads are asymmetric and average the left and right postocular distance. |
| **PrOc** | Preocular distance in lateral view; in Tetramorium, Pheidole and Monomorium: the shortest distance between the anterior eye margin and the sharp frontal margin of the gena. Do not confuse this margin with the edge of the basal mandibular torus. In Messor and Plagiolepis, the shortest distance between the anterior eye margin to that point of the genal margin which is in closest proximity to the dorsal condyle of mandibular joint. |
| **SL** | Maximum straight line scape length (excluding the articular condyle and its neck) as arithmetic mean of both scapes (Fig.378, Fig.379). Lobiform dorsal protrusions near the scape bent are not considered. |
| **sqPDCL to sqPDO** | Principles of recording: the number of pubescence hairs n crossing a measuring line of length L is counted, hairs just touching the line score as 0.5. Mean pubescence distance is then L/n given in µm. Exact counting is only possible with clean surfaces, high-resolution stereomicroscopy at magnifications &gt;=280x and reflection-reduced illumination visualizing the full length of hairs. Surface spots with torn-off pubescence are excluded from counting. |
| **sqPDG** | Square root of pubescence distance PDG in µm on the dorsomedian part of first gaster tergite. In case of strong surface damage or deformation, the second tergite may be used. To reduce accidental errors, several countings along differently positioned, transverse measuring lines are averaged until the sum of hairs counted is 50 at least. |

Supplementary Table 2: Morphometric characters of workers of the cryptic species *Plagiolepis pyrenaica* stat. rev. and *Plagiolepis taurica* given as nest-sample means (n) and individual data (i). The data are allometrically corrected for the assumption of all individuals having CS=450 µm. The columns with p and F values show the results of an univariate ANOVA test with the most separating characters given in heavy type.

|  | ***~~barbara~~ pyrenaica* stat. rev.**  (n=36) | **p**  **F_1,99_** | ***taurica***  (n=64) | ***~~barbara~~ pyrenaica* stat. rev.**  (i=105) | **p**  **F_1,297_** | ***taurica***  (i=194) |
| --- | --- | --- | --- | --- | --- | --- |
| CS [µm] | 463 ± 24  [411,511] | n.s.  2.12 | 455 ± 28  [398,531] | 463 ± 33  [379,558] | n.s.  2.27 | 456 ± 39  [382,559] |
| CL/CW_450_ | 1.133 ± 0.014  [1.113,1.165] | 0.000  **71.57** | 1.111 ± 0.012  [1.088,1.153] | 1.133 ± 0.018  [1.093,1.182] | 0.000  **109.10** | 1.111 ± 0.016  [1.070,1.173] |
| dTP/CS_450_ | 0.520 ± 0.007  [0.508,0.537] | 0.000  **69.12** | 0.508 ± 0.006  [0.490,0.522] | 0.520 ± 0.009  [0.497,0.541] | 0.000  **107.75** | 0.508 ± 0.009  [0.484,0.529] |
| dAN/CS_450_ | 0.254 ± 0.004  [0.240,0.263] | 0.001  11.67 | 0.250 ± 0.006  [0.242,0.264] | 0.254 ± 0.006  [0.232,0.265] | 0.000  19.10 | 0.250 ± 0.007  [0.233,0.268] |
| EL/CS_450_ | 0.282 ± 0.005  [0.269,0.292] | n.s.  3.88 | 0.279 ± 0.008  [0.264,0.301] | 0.282 ± 0.007  [0.266,0.299] | 0.005  7.85 | 0.279 ± 0.009  [0.256,0.309] |
| PrOc/CL_450_ | 0.235 ± 0.007  [0.224,0.248] | 0.000  **42.73** | 0.225 ± 0.008  [0.209,0.244] | 0.235 ± 0.009  [0.209,0.258] | 0.000  **81.59** | 0.225 ± 0.009  [0.199,0.247] |
| PoOc/CL_450_ | 0.364 ± 0.006  [0.348,0.376] | 0.001  11.22 | 0.369 ± 0.008  [0.351,0.391] | 0.364 ± 0.008  [0.341,0.379] | 0.000  19.85 | 0.369 ± 0.010  [0.344,0.396] |
| SL/CS_450_ | 0.935 ± 0.011  [0.907,0.959] | 0.000  **77.21** | 0.911 ± 0.014  [0.881,0.952] | 0.935 ± 0.014  [0.889,0.967] | 0.000  **135.92** | 0.912 ± 0.018  [0.867,0.959] |
| Fu2/CS_450_  [%] | 7.29 ± 0.25  [6.68, 7.79] | 0.000  **56.57** | 6.88 ± 0.27  [6.25, 7.49] | 7.29 ± 0.33  [6.58, 8.15] | 0.000  **88.41** | 6.89 ± 0.35  [5.92, 7.83] |
| Fu3/CS_450_  [%] | 9.13 ± 0.26  [8.47, 9.65] | 0.000  **78.58** | 8.61 ± 0.29  [7.88, 9.30] | 9.12 ± 0.34  [8.09,10.05] | 0.000  **115.14** | 8.63 ± 0.39  [7.57, 9.62] |
| Fu4/CS_450_  [%] | 10.30 ± 0.31  [9.75,10.95] | 0.002  10.00 | 10.08 ± 0.34  [9.42,10.95] | 10.28 ± 0.39  [9.29,11.22] | 0.000  13.01 | 10.10 ± 0.45  [8.50,11.27] |
| Fu4/Fu3_450_ | 1.129 ± 0.037  [1.041,1.210] | 0.000  34.57 | 1.172 ± 0.034  [1.109,1.265] | 1.129 ± 0.050  [1.008,1.294] | 0.000  43.41 | 1.171 ± 0.054  [1.059,1.357] |
| ML/CS_450_ | 1.184 ± 0.026  [1.126,1.242] | 0.000  **54.20** | 1.146 ± 0.024  [1.098,1.206] | 1.182 ± 0.030  [1.121,1.256] | 0.000  **87.73** | 1.147 ± 0.031  [1.068,1.271] |
| MW/CS_450_ | 0.627 ± 0.017  [0.594,0.661] | n.s.  0.45 | 0.625 ± 0.014  [0.585,0.659] | 0.627 ± 0.022  [0.584,0.673] | n.s.  0.24 | 0.625 ± 0.019  [0.571,0.682] |
| PLG/CS_450_  [%] | 10.00 ± 0.27  [9.36,10.58] | 0.000  **56.39** | 9.34 ± 0.48  [8.20,10.42] | 10.00 ± 0.36  [9.03,10.75] | 0.000  **118.93** | 9.35 ± 0.55  [7.95,10.52] |
| sqPDG_450_ | 4.52 ± 0.17  [4.02,4.79] | 0.000  15.67 | 4.69 ± 0.22  [3.98,5.18] | 4.52 ± 0.24  [3.85,5.31] | 0.000  19.69 | 4.67 ± 0.30  [3.51,5.39] |
| BPdG_450_  [µm] | 30.29 ±1.37  [26.2,32.8] | n.s.  0.03 | 30.34 ±1.44  [26.2,33.1] | 30.30 ±1.83  [25.4,35.9] | n.s.  0.09 | 30.23 ±1.97  [23.3,35.8] |

Supplementary Table 3: Morphometric data of worker individuals of the six species of the *Plagiolepis pallescens* group. The data are allometrically corrected for the assumption of all individuals having CS=450 µm.

|  | ***pallescens***  (i=12) | ***pyrenaica* stat. rev.**  (i=105) | ***taurica***  (i=194) | ***minu***  (i=5) | ***sordida***  (i=5) | ***isis***  (i=2) |
| --- | --- | --- | --- | --- | --- | --- |
| CS [µm] | 448 ± 33  [406,502] | 463 ± 33  [379,558] | 456 ± 39  [382,559] | 521 ± 6  [502,541] | 409 ± 36  [376,446] | 420 ± 19  [407,434] |
| CL/CW_450_ | 1.126 ± 0.016  [1.104,1.148] | 1.133 ± 0.018  [1.093,1.182] | 1.111 ± 0.016  [1.070,1.173] | 1.099 ± 0.019  [1.076,1.121] | 1.104 ± 0.012  [1.086,1.111] | 1.096 ± 0.015  [1.085,1.106] |
| dTP/CS_450_ | 0.503 ± 0.009  [0.485,0.521] | 0.520 ± 0.009  [0.497,0.541] | 0.508 ± 0.009  [0.484,0.529] | 0.513 ± 0.012  [0.498,0.526] | 0.510 ± 0.010  [0.500,0.524] | 0.506 ± 0.017  [0.494,0.518] |
| dAN/CS_45_0 | 0.242 ± 0.008  [0.225,0.251] | 0.254 ± 0.006  [0.232,0.265] | 0.250 ± 0.007  [0.233,0.268] | 0.252 ± 0.005  [0.245,0.258] | 0.252 ± 0.002  [0.251,0.256] | 0.252 ± 0.014  [0.242,0.262] |
| EL/CS_450_ | 0.288 ± 0.009  [0.280,0.306] | 0.282 ± 0.007  [0.266,0.299] | 0.279 ± 0.009  [0.256,0.309] | 0.265 ± 0.006  [0.258,0.270] | 0.293 ± 0.003  [0.290,0.298] | 0.268 ± 0.004  [0.265,0.270] |
| PrOc/CL_450_ | 0.240 ± 0.008  [0.229,0.259] | 0.235 ± 0.009  [0.209,0.258] | 0.225 ± 0.009  [0.199,0.247] | 0.252 ± 0.009  [0.239,0.260] | 0.231 ± 0.005  [0.226,0.237] | 0.219 ± 0.007  [0.214,0.224] |
| PoOc/CL_450_ | 0.357 ± 0.009  [0.339,0.373] | 0.364 ± 0.008  [0.341,0.379] | 0.369 ± 0.010  [0.344,0.396] | 0.396 ± 0.011  [0.380,0.407] | 0.354 ± 0.005  [0.349,0.358] | 0.386 ± 0.002  [0.385,0.388] |
| SL/CS_450_ | 1.019 ± 0.031  [0.972,1.060] | 0.935 ± 0.014  [0.889,0.967] | 0.912 ± 0.018  [0.867,0.959] | 0.915 ± 0.017  [0.899,0.939] | 0.919 ± 0.019  [0.901,0.943] | 0.909 ± 0.014  [0.899,0.919] |
| Fu2/CS_450_  [%] | 7.56 ± 0.41  [6.67, 8.15] | 7.29 ± 0.33  [6.58, 8.15] | 6.89 ± 0.35  [5.92, 7.83] | 6.38 ± 0.11  [6.21, 6.48] | 7.46 ± 0.44  [7.13, 8.10] | 8.47 (i=1) |
| Fu3/CS_450_  [%] | 10.21 ± 0.74  [9.15,11.27] | 9.12 ± 0.34  [8.09,10.05] | 8.63 ± 0.39  [7.57, 9.62] | 8.15 ± 0.28  [7.71, 8.42] | 8.46 ± 0.33  [8.16, 8.82] | 9.40 (i=1) |
| Fu4/CS_450_  [%] | 11.69 ± 0.58  [10.71,12.44] | 10.28 ± 0.39  [9.29,11.22] | 10.10 ± 0.45  [8.50,11.27] | 10.09 ± 0.50  [9.54,10.61] | 10.43 ± 0.29  [10.12,10.79] | 10.15 (i=1) |
| Fu4/Fu3_450_ | 1.148 ± 0.056  [1.078,1.249] | 1.129 ± 0.050  [1.008,1.294] | 1.171 ± 0.054  [1.059,1.357] | 1.239 ± 0.038  [1.190,1.292] | 1.232 ± 0.027  [1.192,1.252] | 1.079 (i=1) |
| ML/CS_450_ | 1.212 ± 0.030  [1.173,1.281] | 1.182 ± 0.030  [1.121,1.256] | 1.147 ± 0.031  [1.068,1.271] | 1.120 ± 0.017  [1.099,1.138] | 1.168 ± 0.025  [1.192,1.190] | 1.158 (i=1) |
| MW/CS_450_ | 0.630 ± 0.029  [0.600,0.715] | 0.627 ± 0.022  [0.584,0.673] | 0.625 ± 0.019  [0.571,0.682] | 0.635 ± 0.028  [0.593,0.664] | 0.610 ± 0.003  [0.606,0.613] | 0.628 ± 0.006  [0.624,0.632] |
| PLG/CS_450_  [%] | 9.06 ± 0.54  [7.83, 10.08] | 10.00 ± 0.36  [9.03, 10.75] | 9.35 ± 0.55  [7.95, 10.52] | 8.24 ± 0.11  [8.08, 8.38] | 7.84 ± 0.56  [7.26, 8.57] | 8.34 ± 0.39  [8.06, 8.61] |
| sqPDG_450_ | 4.45 ± 0.28  [4.11,5.13] | 4.52 ± 0.24  [3.85,5.31] | 4.67 ± 0.30  [3.51,5.39] | 5.08 ± 0.25  [4.82,5.39] | 4.16 ± 0.19  [3.94,4.40] | 4.18 ± 0.18  [4.06,4.31] |
| BPdG_450_  [µm] | 28.32 ±2.27  [25.8,34.5] | 30.30 ±1.83  [25.4,35.9] | 30.23 ±1.97  [23.3,35.8] | 30.68 ±1.60  [29.0,32.4] | 24.58 ±1.49  [22.5,25.8] | 25.60 ±0.42  [25.3,25.9] |

Supplementary Table 4: Scores for model performance from three pairwise model comparisons using the 2D dadi pipeline (*P*. *pyrenaica* stat. rev. vs. *P*. *taurica*, and *P*. *pyrenaica* stat. rev. vs. subgroups of *P*. taurica). The best performing models are shown in Figure 4 and corresponding parameter estimates are given in Table 1. Abbreviations: AIC, Akaike information criterion; ΔAIC, difference in AIC to the best-scoring model of the comparison of two particular groups; ωi, Akaike weight.

| ***P*. *pyrenaica* vs. *P*. *taurica*** | |  |  |  |  |
| --- | --- | --- | --- | --- | --- |
| Model | Log-likelihood | AIC | ΔAIC | ωi | Chi-squared |
| **founder_sec_contact_asym_two_epoch** | -486.06 | 988.12 | 0 | 1.00 | 357.4 |
| founder_asym | -504.28 | 1022.56 | 34.44 | 0.00 | 637.86 |
| founder_sym | -510.87 | 1033.74 | 45.62 | 0.00 | 682.62 |
| founder_anc_asym_two_epoch | -527.36 | 1070.72 | 82.6 | 0.00 | 776.27 |
| vic_sec_contact_asym_mig | -559.11 | 1134.22 | 146.1 | 0.00 | 801.77 |
| founder_anc_sym_two_epoch | -579.32 | 1172.64 | 184.52 | 0.00 | 803.21 |
| founder_nomig_admix_first_epoch | -665.29 | 1344.58 | 356.46 | 0.00 | 1527.8 |
| founder_no_mig_two_epoch | -667.91 | 1347.82 | 359.7 | 0.00 | 1771.69 |
| vic_anc_asym_mig | -709.47 | 1434.94 | 446.82 | 0.00 | 1360.45 |

| ***P*. *pyrenaica* vs. *P*. *taurica* Central Asian-Pontic subgroup** | |  |  |  |  |
| --- | --- | --- | --- | --- | --- |
| Model | Log-likelihood | AIC | ΔAIC | ωi | Chi-squared |
| **sec_contact_asym_mig_size** | **-367.7** | **751.4** | **0** | **0.95** | **70.34** |
| asym_mig_size | -370.85 | 757.7 | 6.3 | 0.04 | 81.81 |
| sym_mig_size | -373.81 | 761.62 | 10.22 | 0.01 | 83.11 |
| sec_contact_sym_mig_size | -377.19 | 768.38 | 16.98 | 0.00 | 83.28 |
| sec_contact_asym_mig_size_three_epoch | -401.69 | 821.38 | 69.98 | 0.00 | 160.68 |
| sec_contact_sym_mig | -455.48 | 920.96 | 169.56 | 0.00 | 238.01 |
| sec_contact_asym_mig | -460.24 | 932.48 | 181.08 | 0.00 | 246.2 |
| sec_contact_asym_mig_three_epoch | -466.67 | 947.34 | 195.94 | 0.00 | 262.4 |
| anc_sym_mig_size | -477.32 | 968.64 | 217.24 | 0.00 | 358.22 |
| anc_asym_mig_size | -481.78 | 979.56 | 228.16 | 0.00 | 411.7 |
| asym_mig_twoepoch | -481.88 | 979.76 | 228.36 | 0.00 | 304.78 |
| sym_mig | -513.39 | 1034.78 | 283.38 | 0.00 | 378.18 |
| asym_mig | -513.6 | 1037.2 | 285.8 | 0.00 | 383.42 |
| sym_mig_twoepoch | -513.59 | 1039.18 | 287.78 | 0.00 | 375.25 |
| sec_contact_sym_mig_three_epoch | -518.03 | 1048.06 | 296.66 | 0.00 | 391.01 |
| anc_sym_mig | -525.46 | 1060.92 | 309.52 | 0.00 | 416.14 |
| sec_contact_sym_mig_size_three_epoch | -529.37 | 1074.74 | 323.34 | 0.00 | 400.75 |
| anc_asym_mig | -565.2 | 1142.4 | 391 | 0.00 | 494.25 |
| no_mig_size | -583.09 | 1178.18 | 426.78 | 0.00 | 928.97 |
| no_mig | -656.94 | 1319.88 | 568.48 | 0.00 | 1040.64 |

| ***P*. *pyrenaica* vs. *P*. *taurica* Balkanic-Pannonian subgroup** | |  |  |  |  |
| --- | --- | --- | --- | --- | --- |
| Model | Log-likelihood | AIC | ΔAIC | ωi | Chi-squared |
| **sec_contact_sym_mig_size_three_epoch** | **-536.87** | **1089.74** | **0** | **1.00** | **132.23** |
| sec_contact_asym_mig_size_three_epoch | -543.36 | 1104.72 | 14.98 | 0.00 | 149.75 |
| asym_mig_size | -551.19 | 1118.38 | 28.64 | 0.00 | 170.27 |
| sec_contact_asym_mig_size | -551.21 | 1118.42 | 28.68 | 0.00 | 141.97 |
| sym_mig_size | -556.96 | 1127.92 | 38.18 | 0.00 | 211.3 |
| sec_contact_sym_mig_size | -561.71 | 1137.42 | 47.68 | 0.00 | 257.36 |
| sec_contact_sym_mig | -636.96 | 1283.92 | 194.18 | 0.00 | 412.98 |
| sec_contact_asym_mig | -640.32 | 1292.64 | 202.9 | 0.00 | 413.07 |
| sec_contact_asym_mig_three_epoch | -660.31 | 1334.62 | 244.88 | 0.00 | 554.64 |
| sym_mig_twoepoch | -682.29 | 1376.58 | 286.84 | 0.00 | 559.83 |
| anc_asym_mig_size | -725.92 | 1467.84 | 378.1 | 0.00 | 757.16 |
| sec_contact_sym_mig_three_epoch | -735.45 | 1482.9 | 393.16 | 0.00 | 1026.99 |
| anc_sym_mig_size | -740.72 | 1495.44 | 405.7 | 0.00 | 769.4 |
| asym_mig | -784.81 | 1579.62 | 489.88 | 0.00 | 1075.03 |
| sym_mig | -789.11 | 1586.22 | 496.48 | 0.00 | 1100.26 |
| asym_mig_twoepoch | -789.81 | 1595.62 | 505.88 | 0.00 | 1123.99 |
| anc_sym_mig | -798.65 | 1607.3 | 517.56 | 0.00 | 1208.44 |
| anc_asym_mig | -798.1 | 1608.2 | 518.46 | 0.00 | 1328.82 |
| no_mig | -1041.39 | 2088.78 | 999.04 | 0.00 | 2532.84 |
| no_mig_size | -1044.91 | 2101.82 | 1012.08 | 0.00 | 2324.77 |

Supplementary Table 5. Climatic variables used for ecological niche modelling and their contribution in percent as returned by the niche modelling.

| **Variable** | **Description** | ***Plagiolepis taurica*** | ***Plagiolepis pyrenaica* stat. rev.** |
| --- | --- | --- | --- |
| bio11 | Mean Temperature of Coldest Quarter | 59.493 | 0.168 |
| bio10 | Mean Temperature of Warmest Quarter | 25.910 | 1.176 |
| bio4 | Temperature Seasonality (standard deviation ×100) | 10.633 | 38.848 |
| bio12 | Annual Precipitation | 2.718 | 1.316 |
| bio2 | Mean Diurnal Range (Mean of monthly (max temp - min temp) | 0.814 | 4.014 |
| bio16 | Precipitation of Wettest Quarter | 0.413 | 10.212 |
| bio8 | Mean Temperature of Wettest Quarter | 0 | 7.535 |
| bio17 | Precipitation of Driest Quarter | 0 | 13.723 |
| bio15 | Precipitation Seasonality (Coefficient of Variation) | 0 | 12.981 |
| bio3 | Isothermality (BIO2/BIO7) (×100) | 0 | 8.939 |
| bio9 | Mean Temperature of Driest Quarter | 0 | 1.088 |


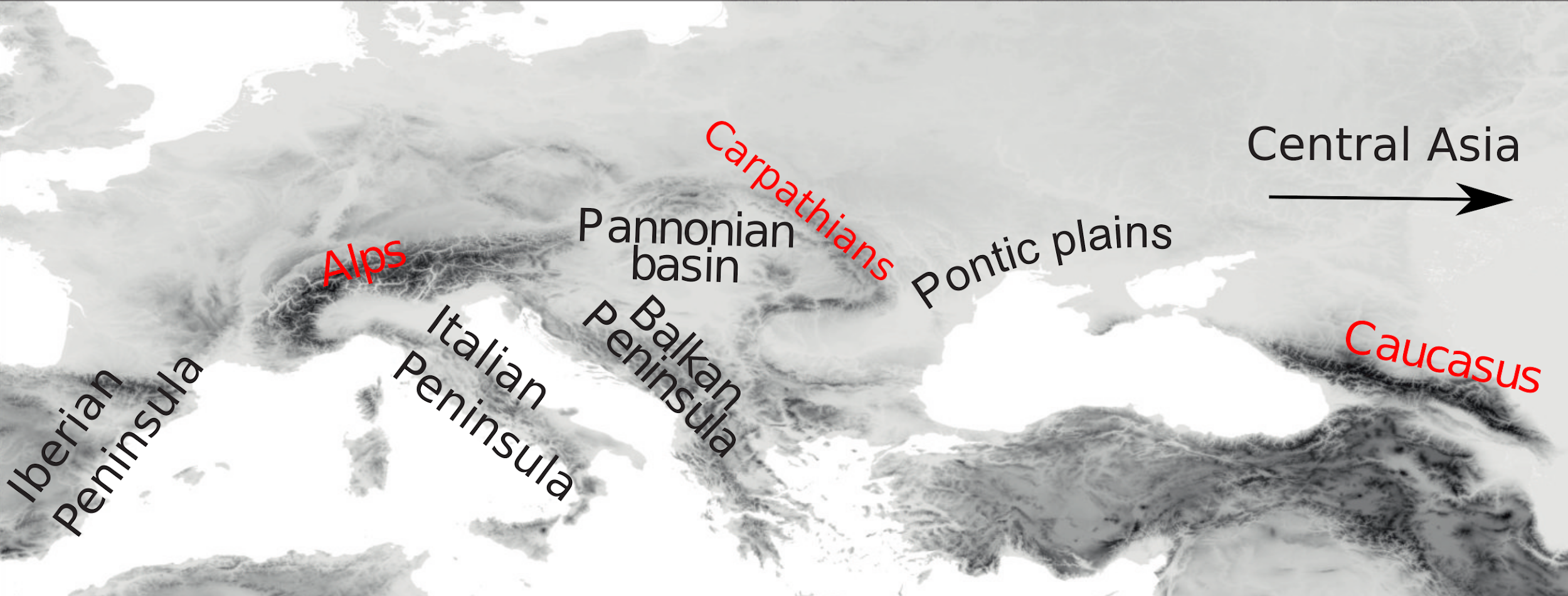
Supplementary Figure 1. Map showing the location of the toponyms as used throughout the manuscript; major European mountain ranges are indicated by red letters.


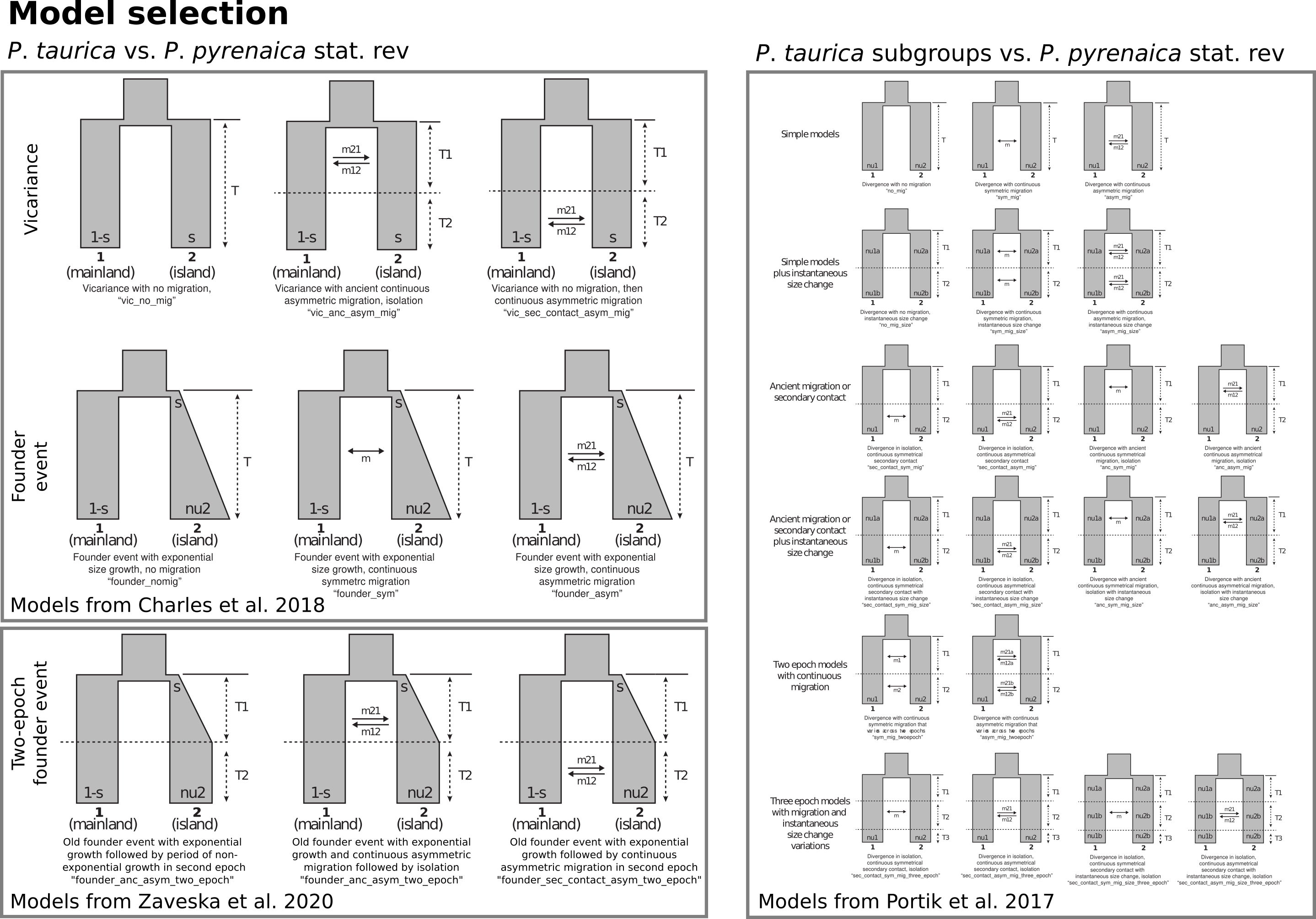
Supplementary Figure 2. Models used for demographic modeling of respective species and lineage pairs.


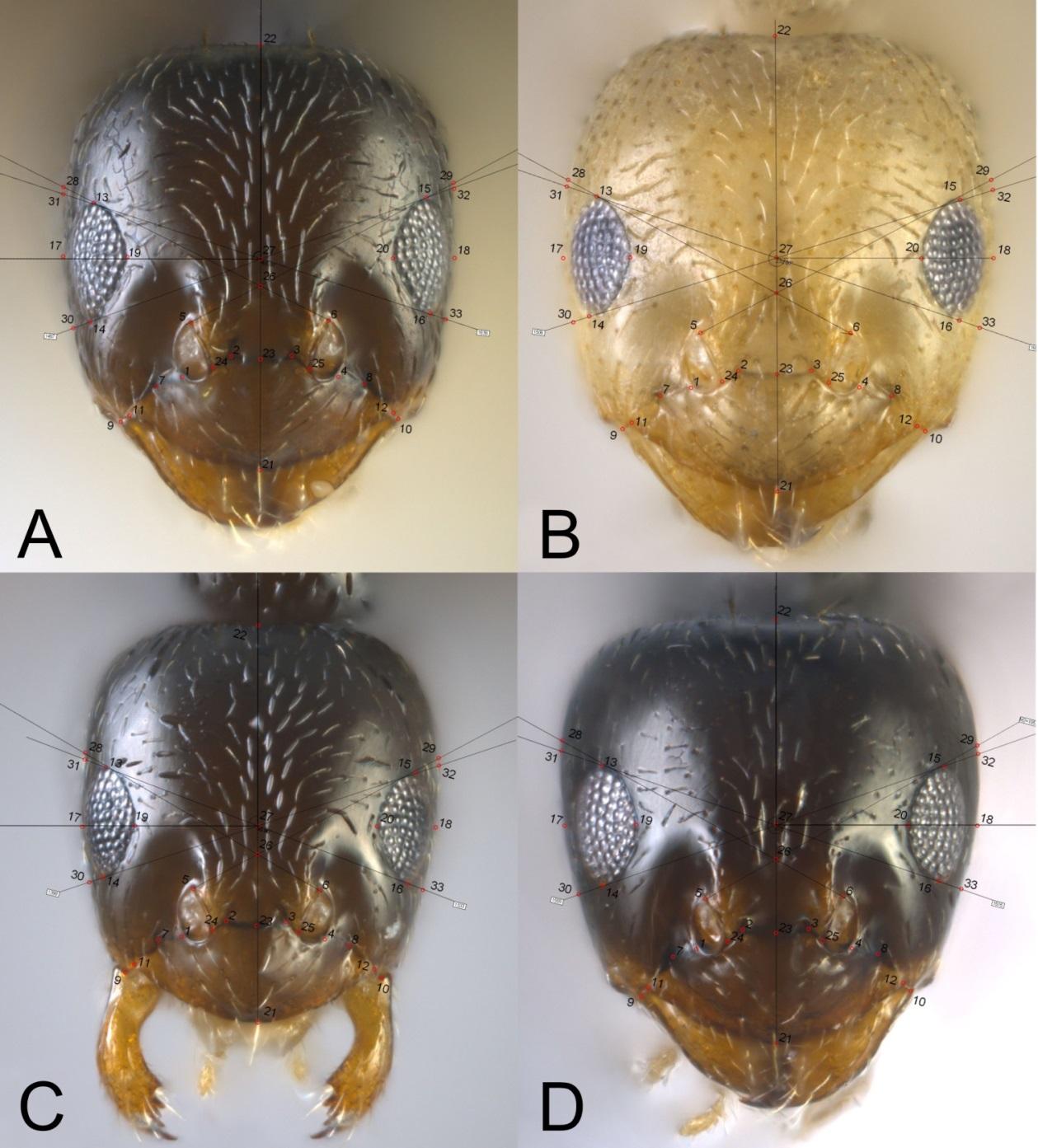
Supplementary Figure 3: Location of the 33 landmarks on *Plagiolepis* heads that were used for geometric morphometrics (GM); **A-D** show heads from specimens from different nests.


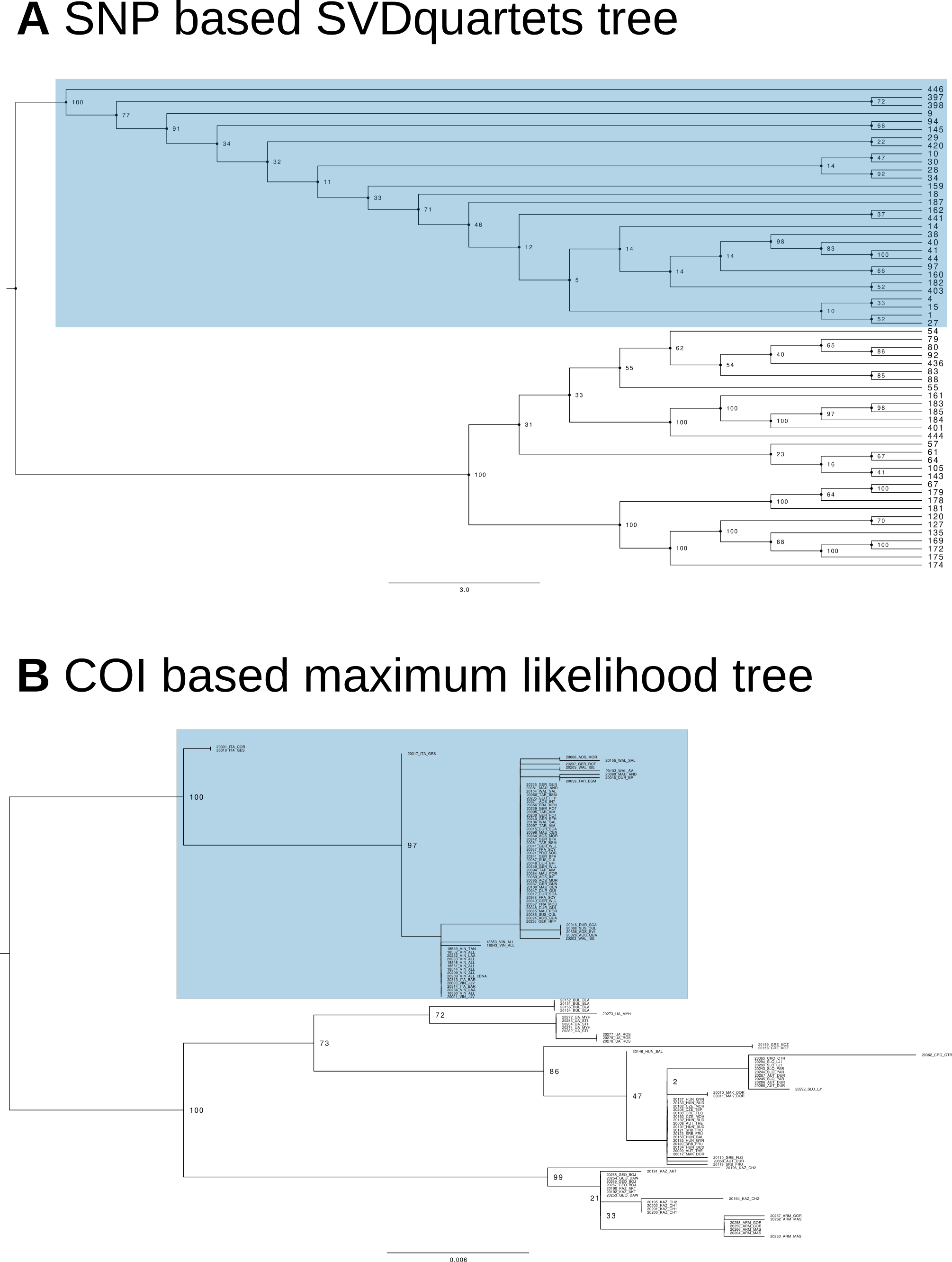
Supplementary Figure 4. Unscaled phylogenetic trees. The blue background marks *P*.  *pyrenaica* stat. rev. as explained in the main text. Node values depict bootstrap support. **A**. SVDquartets tree based on single nucleotide polymorphisms (SNPs) inferred via restriction site associated DNA sequencing (RADseq). **B**. Maximum likelihood tree based on a fragment of the mitochondrial Cytochrome c oxidase subunit 1 gene.


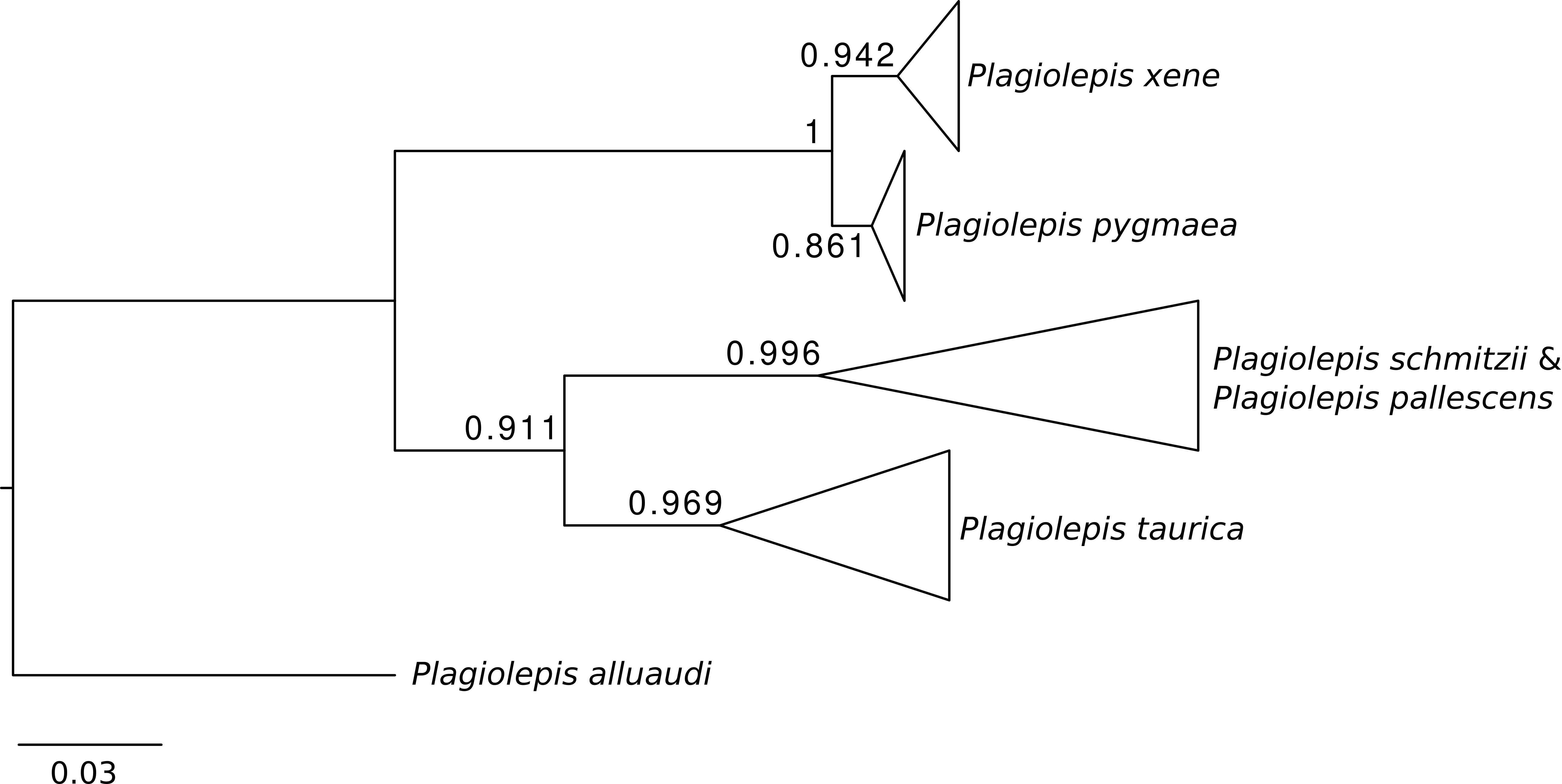
Supplementary Figure 5. Maximum likelihood phylogenetic tree based on available mitochondrial Cytochrome c oxidase subunit 1 sequences from European *Plagiolepis* species. Node values depict bootstrap support.


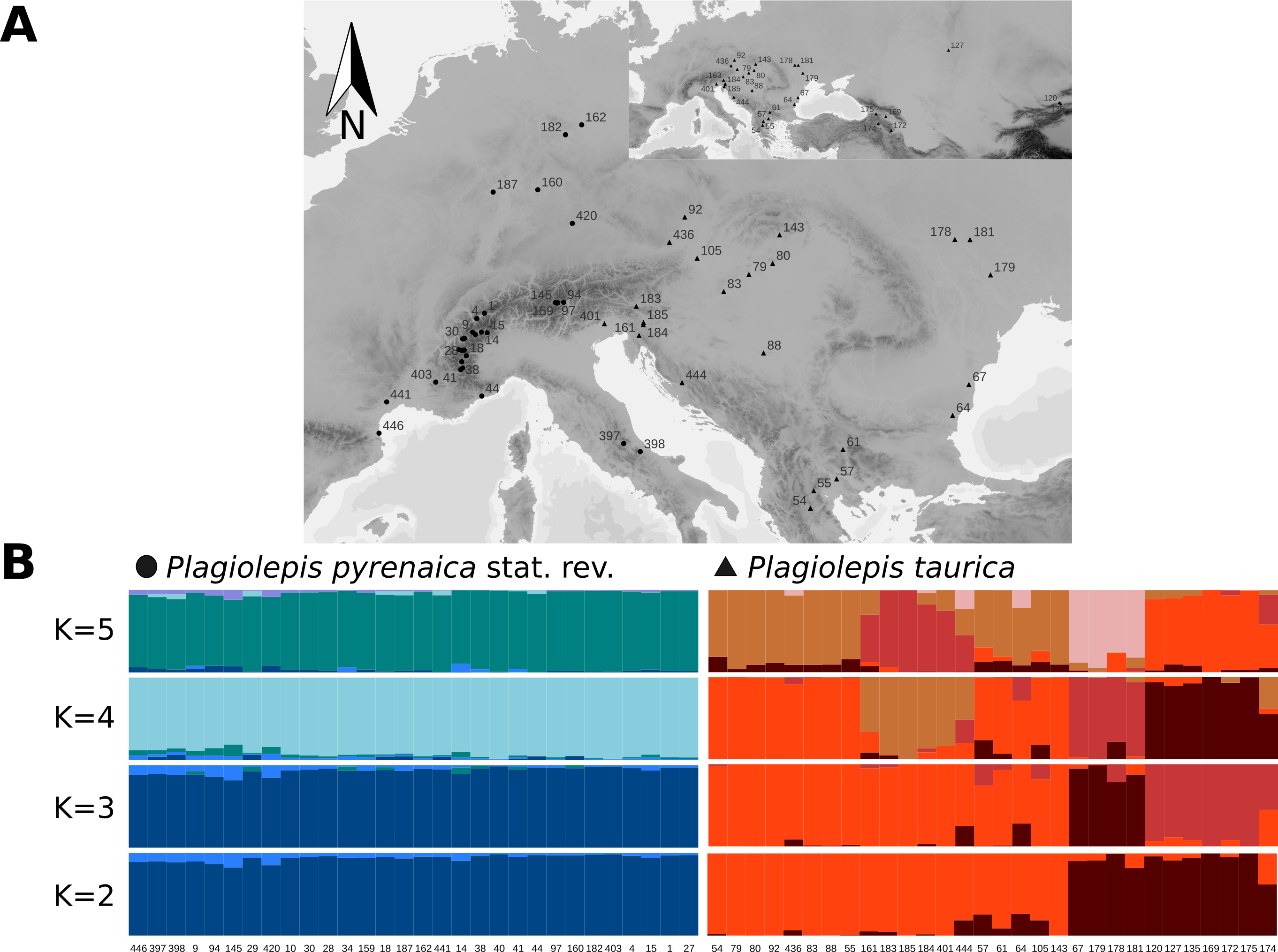
Supplementary Figure 6. **A**. Map showing populations sampled and the corresponding site identification numbers (Site ID in Supplementary Data 1). **B**. Bayesian clustering results of the subgroups obtained via Bayesian clustering of the full dataset assuming K=2 (these two groups also reflect the species hypotheses). Results are shown for K=2 to K=5 clusters. Each barplot represents a population; cluster assignment of each population and proportion of admixture are indicated by colors.


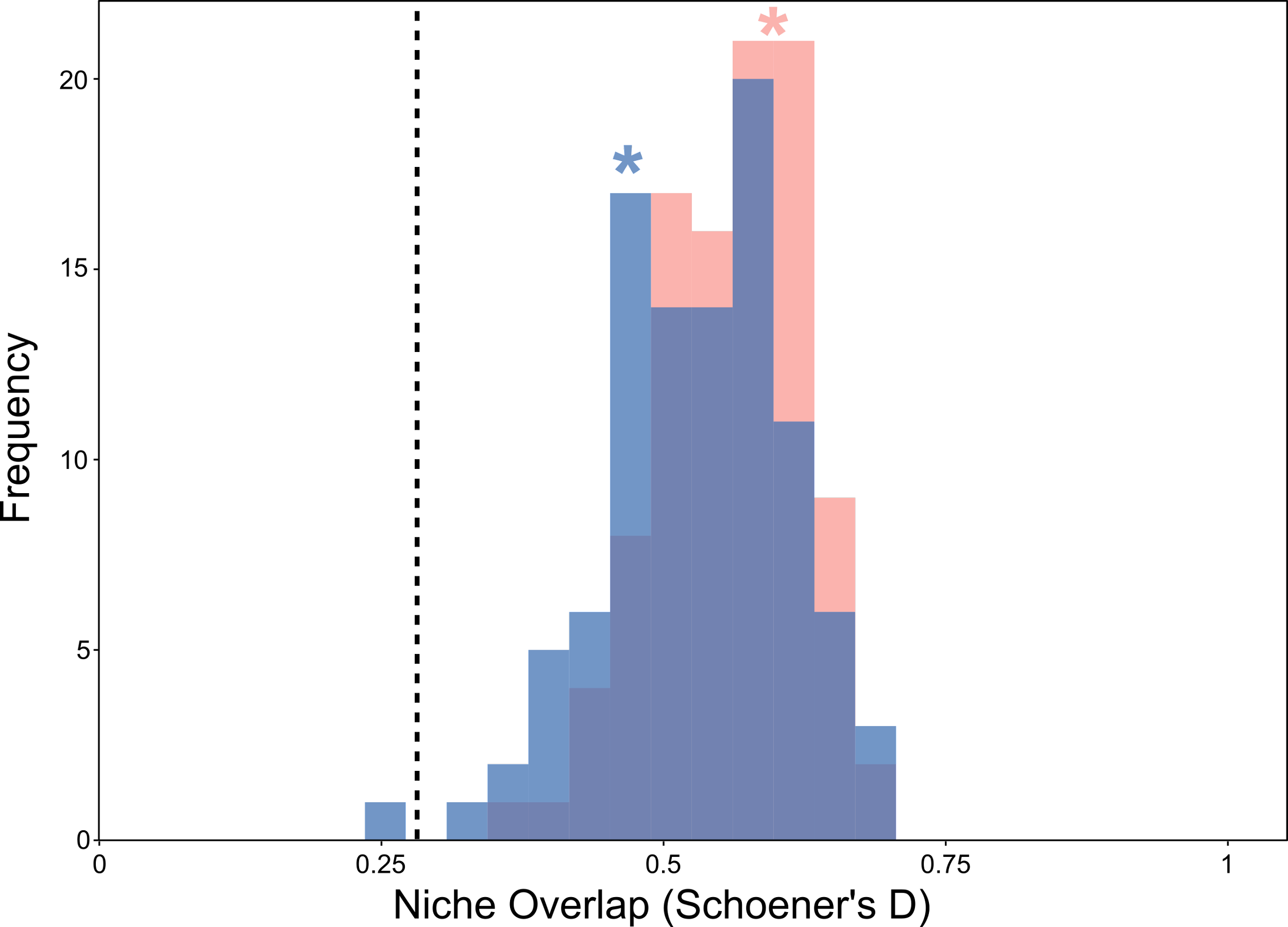
Supplementary Figure 7. Result from pariweise background tests of niche divergence for *Plagiolepis*  *pyrenaica* stat. rev. vs. *Plagiolepis* *taurica* (blue) and vice versa (red). The dashed line marks the empirical niche overlap (Schoener’s *D*) for the pairwise combination of both groups. The coloured bars represent Schoener’s *D* inferred from 100 replicated models per group, each based on randomly sampled background points. Both distributions showed to be significantly more divergent than expected from the empirical overlap (p < 0.05, alpha = 0.05).


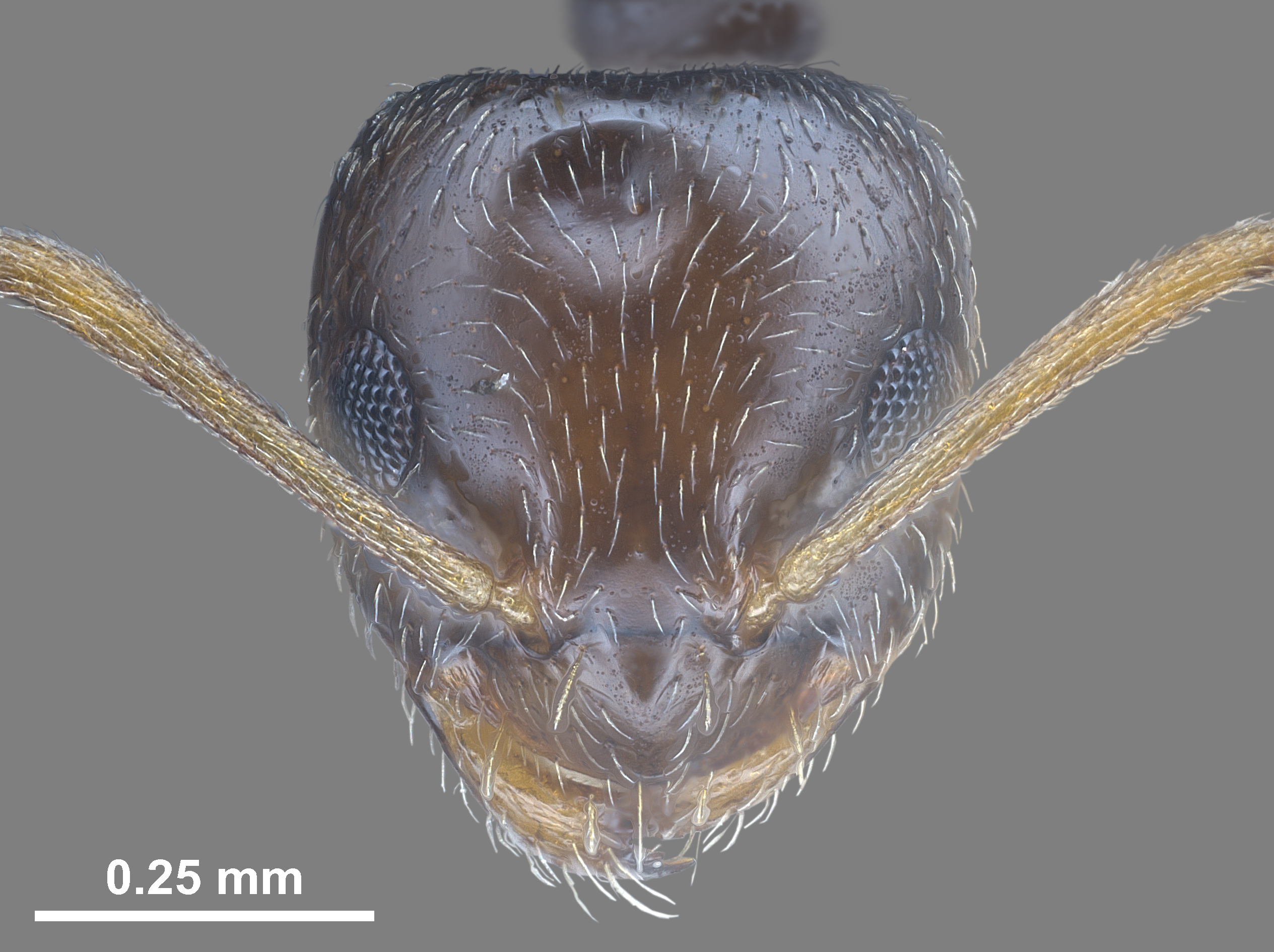
Supplementary Figure 8. Head in dorsal view of a worker of *Plagiolepis* *pyreanaica* stat. rev.


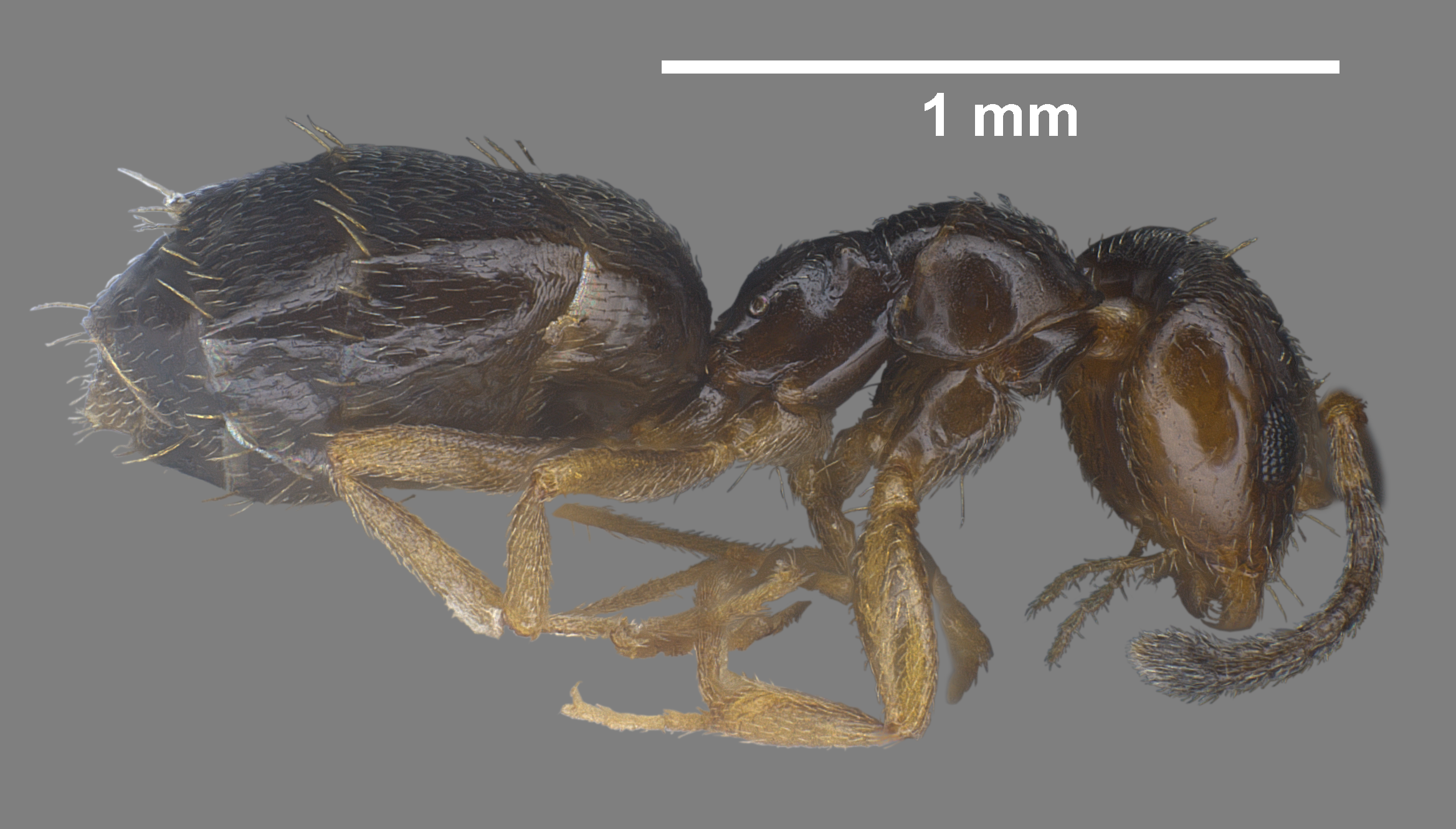
Supplementary Figure 9. Lateral view of a worker of *Plagiolepis* *pyreanaica* stat. rev.
